# Transcriptome-Based Spatiotemporal Analysis of Drought Response Mechanisms in Two Distinct Peanut Cultivars

**DOI:** 10.3390/ijms252211895

**Published:** 2024-11-05

**Authors:** Zexin Sun, Wei Liu, Xinning Wang, Xin Ai, Zhao Li, Dongying Zhou, Qianchi Ma, Yujiao Li, Jiaqi Wang, Xinlei Ma, Xiaoguang Wang, Chao Zhong, Chunji Jiang, Shuli Zhao, He Zhang, Xinhua Zhao, Shuli Kang, Jing Wang, Haiqiu Yu

**Affiliations:** College of Agronomy, Shenyang Agricultural University, Shenyang 110866, China; 13753620538@163.com (Z.S.); lw1456357@163.com (W.L.); wxn20001003@163.com (X.W.); aixin199609@163.com (X.A.); lizhao187@126.com (Z.L.); 2021200081@stu.syau.edu.cn (D.Z.); mqcnihao20000@163.com (Q.M.); 13700157507@163.com (Y.L.); wjq2023240439@163.com (J.W.); maxinlei5966@126.com (X.M.); wxg@syau.edu.cn (X.W.); zhongchao1123@syau.edu.cn (C.Z.); jiangchunji2002@syau.edu.cn (C.J.); zhaoshuli798@syau.edu.cn (S.Z.); zhanghe@syau.edu.cn (H.Z.); xinhua_zhao@syau.edu.cn (X.Z.); kangshuli7004@163.com (S.K.)

**Keywords:** peanut, drought stress, physiology, biochemistry, anatomy, photosynthesis, transcriptome, spatiotemporal analysis

## Abstract

Drought tolerance varies among different peanut (*Arachis hypogaea* L.) cultivars. Here, drought responses of two cultivars, Huayu 22 (HY22) with drought tolerance and Fuhua 18 (FH18) with drought sensitivity, were compared at the morphological, physiological, biochemical, photosynthetic, and transcriptional levels. Drought stress caused wilting and curling of leaves, bending of stems, and water loss in both cultivars. There was an increase in malondialdehyde (MDA) content under prolonged drought stress, more so in FH18. But the levels of reactive oxygen species (H_2_O_2_) and lipid peroxidation were low in HY22. The activities of superoxide dismutase (SOD), peroxidase (POD), and glutathione reductase (GR) were considerably elevated, corresponding with rapid increases in the accumulation of soluble proteins, soluble sugars, and proline. Transcriptional sequencing showed gene expression varied seriously in HY22, which was upregulated in both stems of two cultivars, though downregulation was less pronounced in HY22. KEGG pathway analysis revealed significant enrichment in four leaf and six stem pathways. Additionally, core genes relating to photosynthesis, carbon fixation, proline synthesis, and sucrose and starch synthesis pathways were identified by correlation analysis. Those gene expressions were variously upregulated in stems of two cultivars, especially in HY22, giving a novel view of the shoot as a whole participating in stress response.

## 1. Introduction

In China, peanut is becoming an increasingly prevalent cash and agricultural crop, with an average planting area of 4,739,970 ha from 2020 to 2022. Furthermore, data from the National Bureau of Statistics show that total peanut production has steadily increased from 17,992,700 tons in 2020 to 18,307,800 tons in 2021 and 18,329,500 tons in 2022 (https://www.stats.gov.cn/ accessed on 3 April 2024). The high yield per unit area of peanuts results in a greater net income for farmers than soybeans and other major crops, except cotton [1]. The peanut has a long flowering period, a developed root system, lacks an obvious critical water demand period, and has a high water utilization rate, making it ideal for dry and infertile land [2]. However, under severe drought conditions, insufficient soil water content during the growth and developmental stages can inhibit peanut growth, ultimately reducing yield [3]. In addition, peanuts are prone to aflatoxin contamination during drought conditions, which negatively impacts crop quality [4].

Global warming, including the frequent occurrence of extreme weather and the increase in annual surface temperatures, has significantly increased the occurrence and severity of droughts in China. To date, arid and semiarid areas account for approximately 52.5% of China’s land area [5]. Drought has emerged as the most serious abiotic stressor that affects peanut production and quality [6]. Liaoning is the third-largest peanut-producing region in China and the largest high-quality peanut production and export base in Northeast China [7]. Droughts in Liaoning Province have regional characteristics, and the semi-arid region in western Liaoning, including Fuxin, has experienced severe water shortages at least 18 times from 1951 to 2018 [8,9]. Consequently, elucidating the effects of drought on the morphological, physiological, biochemical, and agronomic characteristics of peanuts has become a research focus.

During drought stress conditions, leaves are the most vulnerable parts of plants [10]. This is because the palisade tissue, sponge tissue, and guard cells in leaves all contain chloroplasts, which are the main action centers for photosynthesis [11]. Plant stems pump water and nutrients and support the roots and leaves, which act as transforming centers for water storage, retention, and transportation [12]. Under drought stress, the parenchyma cells in the stem atrophy, the diameters of the pulp cavity and cell spaces are reduced, the cuticle of the stem prevents the plant from wilting, and the sclerenchyma and wood fiber tissues strengthen the support of the axial organs to alleviate damage [13]. Drought also affects the size and distribution of cells in the epidermal layer, which are in direct contact with the external environment [14]. The pulp has a protective effect on the vascular tissue and is the primary vehicle for water storage in the mid-column [15].

Drought not only causes cell dehydration but also leads to reduced cell turgor pressure, metabolic disorders, changes in membrane permeability, and mechanical damage [16]. When peanuts are under drought stress, reactive oxygen species (ROS) accumulate and react with proteins, lipids, and DNA, which in turn triggers oxidative stress and leads to the impairment of normal cellular functions. Under drought stress, synergistic effects occur between superoxide dismutase (SOD), peroxidase (POD), and other protective substances in plants that can efficiently scavenge ROS and reduce or avoid the damage caused by membrane lipid peroxidation. Peanuts contain organic solutes such as soluble protein (SP), soluble sugars (SS), and proline (Pro), which can regulate the cytoplasmic osmotic potential and protect organelles, proteins, and plasma membranes, thereby reducing damage to peanut plants [17].

To prevent dehydration, plants reduce stomatal conductance, stomatal opening, and transpiration rate, as this helps prevent water loss through the stomata [18]. Photosynthetic physiology is also directly affected by drought. Light energy can be released in three ways: photochemical reactions, heat energy release, and chlorophyll fluorescence release, all of which have competing internal relationships. If the electron transport rate accelerates photochemical reactions, the release of chlorophyll fluorescence and heat energy will be reduced [19]. Almost all changes in photosynthetic status can be detected by changes in chlorophyll fluorescence, which are sensitive to the environment and serve as “intrinsic” characteristics of photosynthesis and are used to investigate the photosynthetic physiological status of plants and drought response mechanisms [20]. Under drought stress, the growth rate of peanuts decreases, and the accumulation rate of photosynthetic products is inversely proportional to the degree of drought stress [21]. For some maize varieties, drought stress can significantly decrease photosynthetic fluorescence parameters, such as the ratio of maximum light quantum efficiency (Fv/Fm) to variable fluorescence decline (Rfd), and increase the non-photochemical burst coefficient (NPQ). This indicates that leaves under drought stress release excess light energy by increasing heat dissipation, ultimately protecting the photosystem from damage [22]. The primary molecular mechanism used by plants in response to drought stress involves the modulation of different metabolic and signal transduction pathways through cellular perception and the transduction of drought signals, thereby altering gene expression [23]. Xu et al. [24] exposed peanut seedlings to drought conditions simulated using 15% PEG-6000. Transcriptome sequencing data revealed a notable enrichment of differentially expressed genes in metabolic pathways, secondary metabolite biosynthesis, flavonoid biosynthesis, and phytohormone signaling pathways.

Previous studies have primarily focused on changes in the leaf and root systems of peanuts under drought stress during the seedling stage. However, few studies have investigated leaf and stem responses to drought stress, particularly the changes in relevant absorption functions at different parts of the leaves and stems. In this study, the effects of drought stress on the stems and leaves of peanut cultivars with and without drought tolerance were assessed, including morphological, physiological, and biochemical differences, alongside the long-distance induction and transmission of stress signaling molecules to elucidate the dynamic responses of peanut seedlings to drought stress. And those results combined with root responses as described in a previous study [25], thereby constructing a work model of whole plant response to drought stress.

## 2. Results

### 2.1. Morphological Characteristics and Water Content of Shoot

**Figure 1:**
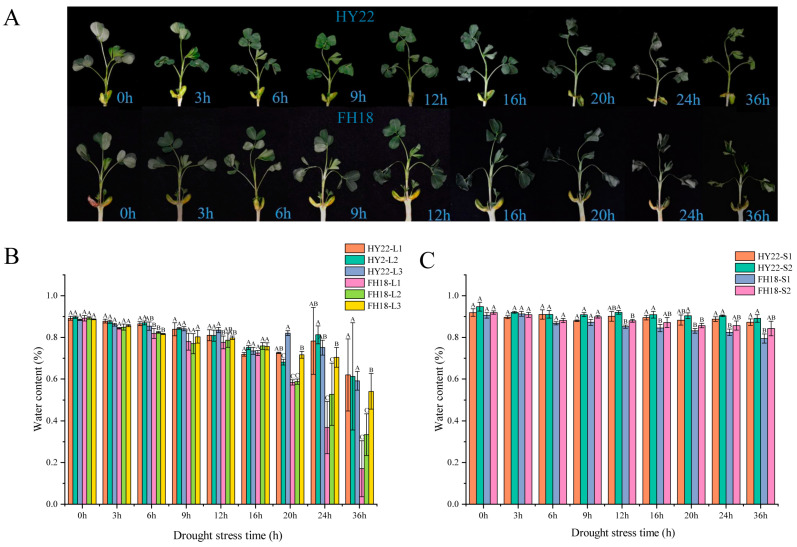
Effects of acute drought stress on the morphology and water content in shoots of different peanut cultivars. (**A**) Morphological changes in the shoots of drought-tolerant cultivar HY22 and the drought-sensitive cultivar FH18 after 0, 3, 6, 9, 12, 16, 20, 24, and 36 h of drought treatment. (**B**) Water content in different layers and (**C**) stem layers from HY22 and FH18 after 0, 3, 6, 9, 12, 16, 20, 4, and 36 h of drought treatment. Values represent the means of three replicates; error bars indicate ± standard error; letters denote significant differences between HY22 and FH18 as determined using analysis of variance (ANOVA; the letters in the graph were significant at the 0.05 level of significance, with significant differences between letters in the same treatment time). L1, L2, and L3 represent different leaf layers from the root to the apical point; S1 and S2 represent different stem levels from the root to the apical point. “HY22” refers to the drought-tolerant cultivar Huayu 22, and “FH18” denotes the drought-sensitive cultivar Fuhua 18.

After 6 h of drought stress, the stems of HY22 began to bend due to lack of water, and L1 began to curl. After 9 h, L2 curled, and after 12 h, L3 curled. The degree of leaf curl gradually increased with drought duration, and the stem shrank owing to excessive water loss (Figure 1A). Overall, the morphological changes in FH18 under stress conditions were more severe than those in HY22. The stems of FH18 lost water and were bent after 3 h, and the time to leaf curl in each layer was shorter in FH18 than that in HY22 (Figure 1B). The results showed that HY22 maintained relatively more erect morphological characteristics than FH18 under drought stress.

Leaf water content in HY22 decreased gradually from 0 to 24 h, and after 24 h, there was a significant decrease in the water content across the three leaf layers; L1 and L2 experienced a slightly faster decline than that experienced by L3. Similarly, the changes in FH18 leaves from 0 to 16 h were mirrored those of HY22, with water content significantly decreasing after 16 h (Figure 1B). The water content of HY22 stems fluctuated and declined from 0 to 12 h of drought treatment; after 12 h, the decline continued but was less pronounced. The overall pattern in stem water content for FH18 was similar to that in HY22, showing fluctuations and a decrease from 0 to 9 h, followed by a more rapid decline after 9 h (Figure 1C). These results showed that FH18 loses water fast in the later stages of drought stress, whereas HY22 had better water retention capacity, which was more conducive to maintaining water potential under drought conditions.

### 2.2. Physiological and Biochemical Characteristics of Peanut Shoots

#### 2.2.1. Membrane and ROS System Damage

**Figure 2:**
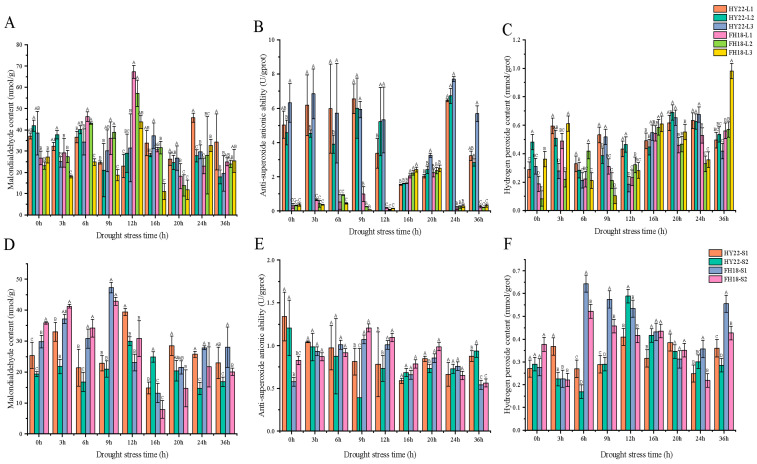
Effects of acute drought stress on malondialdehyde content, anti-superoxide anion activity, and hydrogen peroxide content in the shoots of different peanut cultivars. (**A**) Malondialdehyde content, (**B**) anti-superoxide anion activity, and (**C**) hydrogen peroxide content in different leaf layer samples from the drought-tolerant cultivar HY22 and the drought-sensitive cultivar FH18 after 0, 3, 6, 9, 12, 16, 20, 4, and 36 h of drought treatment. (**D**) Malondialdehyde content, (**E**) anti-superoxide anion activity, and (**F**) hydrogen peroxide content in different leaf layer samples from HY22 and FH18 cultivars after 0, 3, 6, 9, 12, 16, 20, 4, and 36 h of drought treatment. Values are means of three replicates; bars indicate ± standard error; letters indicate significant differences between HY22 and FH18 as determined by analysis of variance (ANOVA; the letters in the graph were significant at the 0.05 level of significance, with significant differences between letters in the same treatment time). L1, L2, and L3 represent different leaf layers from the root to the apical point; S1 and S2 represent different stem levels from the base to the top. “HY22” refers to the drought-tolerant cultivar Huayu 22, and “FH18” denotes the drought-sensitive cultivar Fuhua 18.

Plants produce large amounts of ROS under drought stress and generate malondialdehyde (MDA) through membrane lipid peroxidation [26,27]. Under drought conditions, the MDA contents of the two peanut cultivars first increased and then decreased. A significant MDA peak in HY22 leaves occurred after 24 h, whereas in FH18 leaves, it appeared after 12 h (Figure 2A). The highest MDA peak for HY22 was observed in the stems after 12 h, and for FH18, after 9 h. Thus, significant MDA accumulation occurred earlier in FH18 than that in HY22 (Figure 2D), indicating that HY22 maintained a more intact membrane structure during the early stages of drought stress.

The change in anti-superoxide anion activity was similar to that of superoxide dismutase activity. HY22 leaves peaked after 24 h, whereas FH18 leaves peaked after 16 h (Figure 2B). Similarly, the highest anti-superoxide anion activity peak was observed in the HY22 stems after 36 h, whereas that in the FH18 stems occurred after 9 h (Figure 2E). In addition, the value of HY22 after 24 h was significantly higher than that of FH18, indicating that HY22 was less susceptible to free radical toxicity than FH18. Among the different positions on the stem, it was inferred that the anti-superoxide anion activity was more durable in HY22 cells, which functioned after 36 h.

The hydrogen peroxide content in the leaves of HY22 peaked after 20 and 24 h, while it was significantly higher in FH18 after 3, 16, and 36 h (Figure 2C). Compared with the leaves, the changes in the stems were more pronounced. The peak hydrogen peroxide content in HY22 stem occurred after 12 h, compared to 6 h in FH18 (Figure 2F). Early in the acute drought stress, anti-superoxide anion activity was enhanced in the susceptible cultivar FH18. Conversely, in HY22, the peak of hydrogen peroxide occurred later, indicating that this cultivar exhibited superior anti-superoxide anion activity compared to the drought-sensitive cultivar.

#### 2.2.2. Antioxidant Enzyme

**Figure 3:**
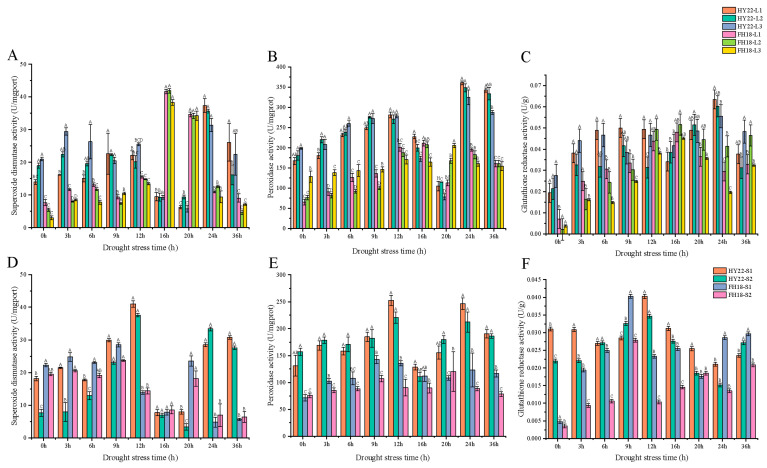
Effects of acute drought stress on superoxide dismutase, peroxidase, and glutathione reductase expression in the shoots of different peanut cultivars. (**A**) Superoxide dismutase, (**B**) peroxidase, and (**C**) glutathione reductase levels in different leaf layer samples from the drought-tolerant cultivar HY22 and the drought-sensitive cultivar FH18 after 0, 3, 6, 9, 12, 16, 20, 4, and 36 h of drought treatment. (**D**) Superoxide dismutase, (**E**) peroxidase, and (**F**) glutathione reductase levels in different stem layer samples from HY22 and FH18 cultivars after 0, 3, 6, 9, 12, 16, 20, 4, and 36 h of drought treatment. Values are means of three replicates; bars indicate ± standard error; letters indicate significant differences between HY22 and FH18 as determined via analysis of variance (ANOVA; the letters in the graph were significant at the 0.05 level of significance, with significant differences between letters in the same treatment time). L1, L2, and L3 represent different leaf layers from the root to the apical point; S1 and S2 represent different stem levels from the root to the apical point.

Superoxide dismutase is an antioxidant enzyme that removes excess ROS produced under stress conditions. Superoxide dismutase activity in HY22 leaves fluctuated and increased from 0 to 24 h of the drought stress. After 24 h, the activity in all leaf layers peaked, with the highest activity observed in L1. Activity levels in L1, L2, and L3 then significantly decreased (Figure 3A). The overall change in FH18 leaves was similar to that in HY22 leaves, and the peak activities of L1 and L2 were observed after 16 h. The turning points in the activity levels in the stems of HY22 and FH18 were significant and occurred at 12 h; however, the change was lower in FH18 (Figure 3D). The peak superoxide dismutase activity in HY22 was significantly higher than that in FH18, enhancing its ability to scavenge excess ROS and thus providing strong protection against drought. Peroxidases, which are oxidoreductases that remove hydrogen peroxide, phenols, and amines, showed a notable increase in HY22 leaves at 24 h. Although the FH18 also showed a significant increase at 16 h, it was considerably lower than that in HY22 at the same time point (Figure 3B). In particular, during the first 3–12 h, peroxidase activity was significantly higher in HY22 than that in FH18, and this trend became even more pronounced after 24 h. The overall change in peroxidase activity in HY22 stem tissues consistently exceeded that in FH18, except at 16 h, with the peak activity occurring at 12 h in HY22 and leaves and at 9 h in FH18, which was significantly lower than that in HY22 (Figure 3E). Generally, both in stems and leaves, HY22 exhibited higher peroxidase activity than that exhibited by FH18, indicating a superior and more sustained protective mechanism.

Glutathione reductase is a key enzyme in the AsA-GSH cycle that primarily uses NADPH to reduce GSSG. Glutathione reductase activity in the leaves of HY22 peaked at 24 h, and the activity level was higher than that of FH18 leaves at the same time, which helped maintain a high GSH/GSSG ratio (Figure 3C). However, it was observed earlier in the leaves of FH18 plants at 16 h and decreased thereafter (Figure 3C). The activity in the stem of HY22 peaked at 12 h and then decreased but was significantly higher than that in FH18 (Figure 3F). Thus, HY22 was equipped with a better antioxidant enzyme system.

#### 2.2.3. Osmotic Adjustments

**Figure 4:**
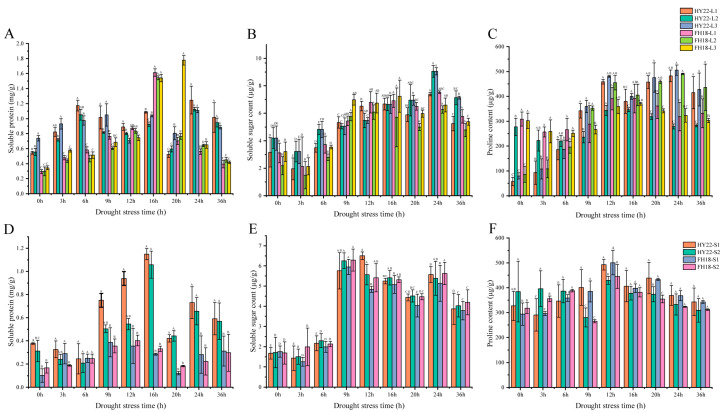
Effects of acute drought stress on soluble protein, soluble sugar, and proline content in the shoots of different peanut cultivars. (**A**) Soluble protein, (**B**) soluble sugar, and (**C**) proline contents in different leaf layer samples from the drought-tolerant cultivar HY22 and the drought-sensitive cultivar FH18 after 0, 3, 6, 9, 12, 16, 20, 4, and 36 h of drought treatment. (**D**) Soluble protein, (**E**) soluble sugar, and (**F**) proline contents in different stem layer samples from HY22 and FH18 cultivars after 0, 3, 6, 9, 12, 16, 20, 4, and 36 h of drought treatment. Values are means of three replicates; error bars indicate ± standard error; letters indicate significant differences between HY22 and FH18 as determined via analysis of variance (ANOVA; the letters in the graph were significant at the 0.05 level of significance, with significant differences between letters in the same treatment time). L1, L2, and L3 represent different leaf layers from the root to the apical point; S1 and S2 represent different stem layers from the root to the apical point.

Soluble proteins, soluble sugars, and proline are osmoregulatory substances significantly induced by drought stress. The SP content of HY22 leaves peaked at 24 h, whereas the L1 and L2 levels of FH18 peaked at 16 h (Figure 4A). The SP content in the stems of HY22 was significantly higher than that of FH18, especially at 9–36 h. The peak activity for HY22 was observed at 16 h, whereas for S1 and S2 of FH18, it was observed at 9 and 16 h, respectively (Figure 4D). Additionally, the SP content in HY22 reached a higher peak than that in FH18, indicating more substantial accumulation of osmotic regulation substances in HY22. Under drought stress conditions, the soluble sugar content in HY22 leaves increased from 0 to 24 h, peaking at 24 h, before decreasing at 36 h. In FH18 leaves, soluble sugar content fluctuated and increased from 0 to 16 h, peaked at 16 h, and then declined from 20 to 36 h (Figure 4B). The accumulation of soluble sugars in S1 and S2 of HY22 peaked at 12 and 9 h, respectively, and fluctuated in later stages. The stems of FH18 peaked at 9 h, followed by a pattern of decreased change in the late stages that closely mirrored the trend observed in HY22 (Figure 4E). The proline content of HY22 leaves increased continuously from 0 to 12 h under drought stress, began to decrease at 16 h, showed a fluctuating trend in later periods, and then peaked at 24 h. The initial proline content of FH18 was higher than that of HY22; however, under drought stress, it showed a trend of first decreasing, then increasing, and then decreasing again, reaching a peak at 16 h (Figure 4C). The changes in proline content in the stems and leaves of both cultivars were similar. The stems of HY22 showed a general trend of first increasing and then decreasing, and then peaked at 12 h, whereas FH18 first decreased, then increased, and then decreased, which was consistent with the time point of the peak for HY22 (Figure 4F). The proline content in the leaves and stems of HY22 increased sharply with the duration of drought stress and was always higher than that in FH18 from 9 to 36 h. These results show that the drought-resistant cultivar HY22 accumulated more osmotic adjustment substances under acute drought stress than the drought-sensitive cultivar FH18.

### 2.3. Anatomical Structures of the Peanut Shoots

**Figure 5:**
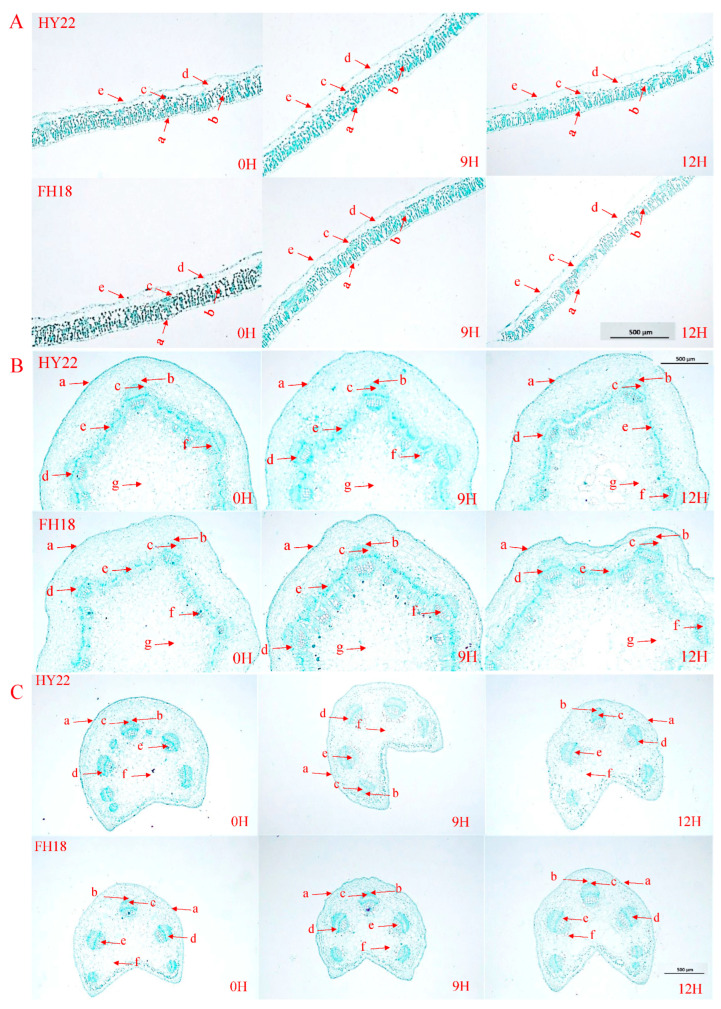
Anatomical structures in different peanut cultivars under acute drought stress at the seedling stage (500×). (**A**) Leaf anatomical structures: a. upper epidermis; b. fence tissue; c. sponge tissue; d. water storage tissue; and e. epidermis. (**B**) Stem anatomical structures: a. epidermis; b. vascular bundle sheath; c. vascular bundle cap; d. phloem; e. cambium; f. xylem; and g. medullary ray. (**C**) Petiole anatomical structures: a. epidermis; b. vascular bundle sheath; c. vascular bundle cap; d. phloem; e. xylem; and f. medullary ray. “HY22” refers to the drought-tolerant cultivar Huayu 22, and “FH18” denotes the drought-sensitive cultivar Fuhua 18. “0H” denotes 0 h after acute drought stress, “9H” denotes 9 h after, and “12H” denotes 12 h after.

Under drought stress, peanut leaves undergo structural changes to adapt to water-deficient environments. The transverse structures of the peanut seedling leaves from the outside to the inside are as follows: upper epidermis, palisade tissue, spongy tissue, water storage tissue, and lower epidermis. In the control (CK), all of the HY22 and FH18 leaf cells were both arranged in a long columnar shape. After 9 h of drought stress, leaf cells were more closely arranged, and their shape did not change significantly. After 12 h of acute drought stress, the cells were more loosely arranged and shortened, and the changes in FH18 were more evident than those in HY22 (Figure 5A). Drought stress had no significant effects on the epidermal structures and water storage tissues of peanut leaves but had significant effects on leaf thickness, palisade tissue thickness, sponge tissue thickness, and the palisade tissue/sponge tissue ratio (Table 1, Figure 5A). Compared with CK, HY22 leaf thickness had decreased by 4.84% and 10.55% after 9 and 12 h of acute drought stress, respectively, whereas that of FH18 decreased by 9.77% and 18.15%, respectively. The leaf thicknesses of HY22 and FH18 were not significantly different after 9 h of drought stress but were significantly different after 12 h. The variation in blade thickness in FH18 was more obvious, indicating that HY22 was better at maintaining water potential (Table 1, Figure 5A). Compared with FH18, HY22 had more developed palisade and spongy tissues (Figure 5A). Under drought stress, the palisade tissue thicknesses of HY22 and FH18 first increased and then decreased, whereas the sponge tissue thicknesses first decreased and then increased (Figure 5A). Compared with the control, the ratio of palisade tissue to spongy tissue in HY22 had increased by 27.59% and 16.81% after 9 and 12 h, respectively, whereas that in FH18 increased by 18.67% and 8.30%, respectively. The results showed that the ratio of palisade to spongy tissue increased significantly in HY22, reflecting its stronger adaptability to arid environments (Table 1; Figure 5A).

The transverse structures of peanut seedling stems, from outside to inside, are arranged as follows: epidermis, vascular bundle sheath, vascular bundle cap, phloem, cambium, xylem, and pith. In CK peanut stems, which were not subjected to drought stress, the epidermis, vascular bundle sheath, and vascular bundle cap structures were well defined and intact (Figure 5B). After 9 h of drought stress, the epidermis of HY22 began to shrink, and the pith structure near the cambium collapsed because of water loss and shrinkage of the peanut stems. The epidermis of FH18 was also crumpled, and the pulp structure near the cambium was destroyed. At the same time, the xylem and cambium began to break, and the degree of damage was more severe than that in HY22 (Figure 5B). After 12 h of drought stress, the epidermal structure of HY22 was similar to that at 9 h; the peripheral structure of the vascular bundle sheath was blurred, the hole in the pulp structure increased, and overall, the damage was more serious than that at 9 h. The epidermal structure of FH18 was folded, the degree of shrinkage was enhanced, and the pulp structure between the epidermis and cambium was damaged. Due to excessive water loss, the structure was folded, the structure of the vascular bundle sheath was cracked, and the structure of the vascular bundle cap was broken. The xylem and cambium were broken, and the pulp was hollow (Figure 5B). Compared with FH18, HY22 maintained a relatively complete structure and supported plants in their functions of transporting nutrients through the stem and carrying out normal life activities.

The transverse structure of the peanut seedling petiole from outside to inside is as follows: epidermis, vascular bundle sheath, vascular bundle cap, phloem, xylem, and pith. The epidermis is a regular layer of closely arranged cells. In the CK, the epidermis of HY22 and FH18 plants appeared flat (Figure 5C). After 9 h of drought stress, the epidermal structure of HY22 was depressed by water loss, but the structures of the bundle sheath and bundle cap did not change significantly. The structural changes in FH18 were similar to those in HY22, including epidermal depression that was greater than that in HY22. Under drought stress, the peripheral structure of the vascular bundle sheath became blurred (Figure 5C). After 12 h of drought stress, the peripheral structure of the bundle sheath observed in HY22 began to crack. The petiole structure of FH18 was severely damaged, the vascular sheath and xylem edges were blurred, and the pulp was in the center of the petiole and comprised a large number of parenchyma cells. At this time, many cells in the pulp began to disassemble (Figure 5C).

### 2.4. Chlorophyll Fluorescence Analysis

**Figure 6:**
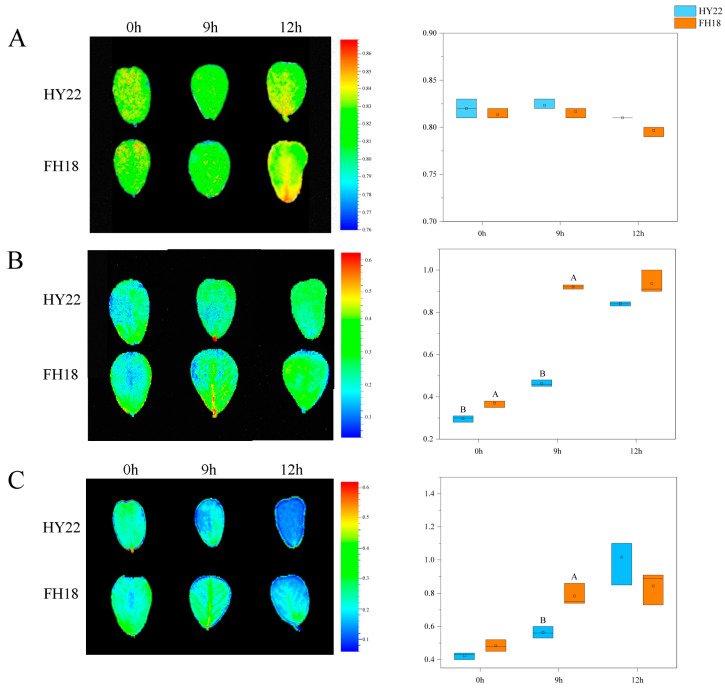
Effects of acute drought stress on chlorophyll fluorescence parameters of the leaves of different peanut cultivars. (**A**) Ratio of maximum light quantum efficiency (Fv/Fm) of L3 leaves from HY22 and FH18 (h). (**B**) Non-photochemical burst coefficient (NPQ) of L3 leaves from HY22 and FH18 (h). (**C**) Variable fluorescence decline (Rfd) in L3 leaves from HY22 and FH18 (h). Data are shown for each panel after 0, 9, and 12 h of drought treatment. Values are means of three replicates; bars indicate ± standard error; and letters indicate significant differences between HY22 and FH18 via analysis of variance (ANOVA; the letters in the graph were significant at the 0.05 level of significance, with significant differences between letters in the same treatment time). “HY22” refers to the drought-tolerant cultivar Huayu 22, and “FH18” to the drought-sensitive cultivar Fuhua 18.

The Fv/Fm ratio represents the photochemical reaction efficiency at the center of PSII. Initially, the leaves of HY22 were yellow at 0 h but changed to yellow-green after 9 h of drought stress. By 12 h, the leaves displayed alternating yellow-green colors (Figure 6A). The trend in leaf color change for FH18 was comparable to that in HY22, except that the yellow occupied a larger proportion of the leaf color in FH18 after 12 h, and the value decreased without significance (Figure 6A).

NPQ can protect the photosynthetic apparatus to a certain extent and acts as a self-protection mechanism in plants. Under drought stress, the leaf colors of the two varieties gradually changed from blue to green, and NPQ showed an upward trend with continued stress (Figure 6B). During the initial 0–12 h of drought stress, the non-photochemical quenching mechanisms of HY22 and FH18 played increasingly significant roles in alleviating the damage caused by drought stress (Figure 6B). During the stress process, the non-photochemical quenching coefficient of FH18 was consistently higher than that of HY22, particularly at 9 h, indicating that drought caused greater damage to FH18 (Figure 6B).

Rfd reflects the photosynthetic potential of plant leaves. Under stress conditions, the leaf color of the two varieties gradually changed from green to blue. During the initial 0–12 h of drought stress, the Rfd values of HY22 and FH18 increased, with the photosynthetic potentials of both varieties increasing to a certain extent. The increasing trend of HY22 was significantly faster than that of FH18, indicating that the photosynthetic potential of HY22 was greater under drought stress (Figure 6C).

### 2.5. Transcription Analysis of Peanut Shoots

#### 2.5.1. RNA Sample Quality Monitoring

Transcriptome sequencing was performed using the leaves and stems of two peanut varieties after 0, 9, and 12 h of drought stress. The test results are presented in Supplementary Table S1. All indicators of the 36 samples met the sequencing standard, and the test results were class A, meeting the requirements for database construction.

#### 2.5.2. Analysis of Differential Gene Expression

A large number of genes were differentially expressed in HY22 and FH18 leaves after 9 h of drought stress (Supplementary Figure S1A). Specifically, 750 and 790 genes were upregulated in HY22 and FH18, respectively, whereas 1478 and 1385 genes were downregulated (Supplementary Figure S1C,D,F,G). In HY22, 14,261 and 12,002 genes were differentially expressed after 9 and 12 h of drought stress, respectively, with 4639 and 3975 genes upregulated and 9622 and 8027 genes downregulated (Supplementary Figure S1A). The numbers of upregulated and downregulated genes in HY22 leaves after 9 h of drought stress were 1.17 and 1.20 times higher, respectively, than those at 12 h. Similarly, after 9 and 12 h of drought stress, 14,089 and 12,729 genes were differentially expressed in the leaves of FH18, with 4728 and 4475 being upregulated and 9361 and 8254 being downregulated, respectively (Supplementary Figure S1A). The upregulated and downregulated gene expression levels in FH18 leaves after 9 h were 1.06 and 1.13 times higher, respectively, than those after 12 h. The results indicate that under drought stress, the degree of gene expression change was more severe in HY22 leaves than that in FH18 leaves, regardless of the upregulation or downregulation of gene expression.

After 9 h of drought stress, numerous genes were differentially expressed in the stems of HY22 and FH18 (Figure 3A). A total of 506 and 435 genes were upregulated in HY22 and FH18 stems, and 725 and 986 genes were downregulated, respectively (Supplementary Figure S1I,J,L,M). After 9 and 12 h of drought stress, 9090 and 8853 genes were differentially expressed in the stems of HY22, among which 3918 and 3207 genes were upregulated and 5172 and 5646 genes were downregulated, respectively (Supplementary Figure S1A). The numbers of upregulated and downregulated genes in the stems of HY22 after 9 h of drought stress were 1.22 and 0.92 times higher than those at 12 h, respectively. After 9 and 12 h of drought stress, 10,537 and 9373 genes were differentially expressed in the stems of FH18, among which 3410 and 2823 genes were upregulated and 7127 and 6550 genes were downregulated, respectively (Supplementary Figure S1A). The numbers of upregulated and downregulated genes in FH18 stems after 9 h of drought stress were 1.21 and 1.09 times greater than those at 12 h, respectively. The results showed that under drought stress, the upregulated gene expression factors in the stems of HY22 were the same as those in FH18, whereas the downregulated gene expression factors in the stems of FH18 were more pronounced than those in the stems of HY22. Under drought stress, the stems and leaves of the different varieties showed differences in their differential gene expression times, indicating that there were differential regulatory mechanisms in the stems and leaves.

### 2.6. Kyoto Encyclopedia of Genes and Genomes (KEGG) Enrichment Analysis of Differentially Expressed Genes

The DEGs in peanut leaves and stem tissues under different drought stress conditions were determined to be enriched in specific pathways based on a *p*-value < 0.05. The results showed that 3190 and 2611 genes were enriched in 134 and 135 pathways in HY22 leaves after 9 and 12 h of drought stress, respectively, including significant enrichment in 56 and 58 metabolic pathways, respectively. After 9 and 12 h of drought stress, 3126 and 2731 FH18 genes were enriched in 137 and 133 pathways, respectively, including significant enrichment in 56 and 60 metabolic pathways, respectively.

There were 133 and 134 pathways with 1842 and 1853 enriched genes in the stems of HY22 plants after 9 and 12 h of drought stress, respectively, of which 44 metabolic pathways were significantly enriched. There were 137 and 135 pathways with 2170 and 1903 enriched genes in FH18 stems after 9 and 12 h of drought stress, respectively, and 44 and 30 metabolic pathways were significantly enriched, respectively.

**Figure 7:**
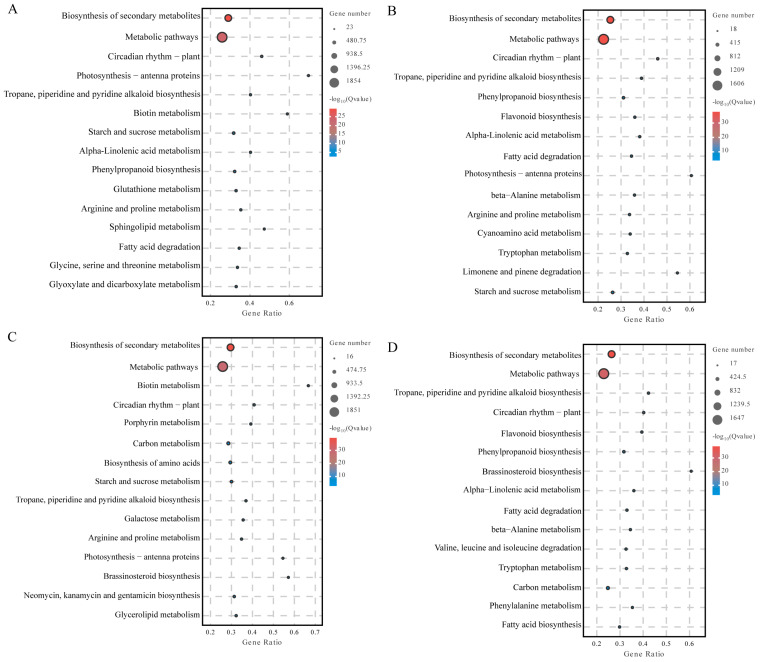
KEGG enrichment analyses of leaves during the seedling stage under acute drought stress. This analysis includes drought-resistant cultivar Huayu 22 (HY22) and drought-sensitive cultivar Fuhua 18 (FH18). For HY22 leaf, comparisons are shown for (**A**) 9 h versus 0 h and (**B**) 12 h versus 0 h after drought initiation. For FH18 leaf, comparisons are shown for (**C**) 9 h versus 0 h and (**D**) 12 h versus 0 h.

Among the first 15 metabolic pathways that were significantly enriched, the differentially expressed genes in HY22 leaves under drought stress were in the metabolic pathways (9 h: 1854/12 h: 1606), biosynthesis of secondary metabolites (9 h: 1142/12 h: 998), carbon metabolism (9 h: 223/12 h: 190), starch and sucrose metabolism (9 h: 147/12 h: 122), plant-pathogen interactions (9 h: 143/12 h: 134), pyruvate metabolism, glycolysis/gluconegenesis (9 h: 112/12h: 100), phenylpropanoid biosynthesis (9 h: 109/12 h: 105), and amino sugar and nucleotide sugar metabolism (9 h: 97/12 h: 92) pathways (Figure 7A,B). The differentially expressed genes in FH18 leaves under drought stress were in the metabolic pathway (9 h: 1849/12 h: 92:1038) and secondary metabolites (9 h: 1164/12 h: 1647), carbon metabolism (9 h: 244/12 h: 69), phenylpropanoid biosynthesis (9 h: 96/12 h: 116), plant circadian rhythm (9 h: 71/12 h: 153), and fatty acid metabolism (9 h: 71/12 h: 87) were significantly enriched in the metabolic pathways (Figure 7C,D).

**Figure 8:**
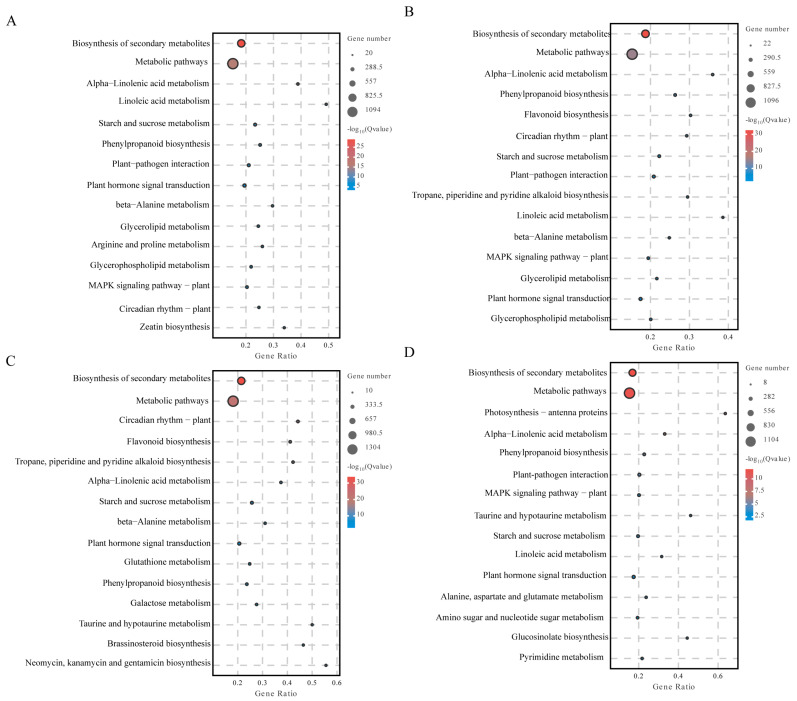
KEGG enrichment analyses of stems during the seedling stage under acute drought stress. This analysis includes drought-resistant cultivar Huayu 22 (HY22) and drought-sensitive cultivar Fuhua 18 (FH18). For HY22 stem, comparisons are shown for (**A**) 9 h versus 0 h and (**B**) 12 h versus 0 h after drought initiation. For FH18 stem, comparisons are shown for (**C**) 9 h versus 0 h and (**D**) 12 h versus 0 h.

Biosynthesis of secondary metabolites in the stems of HY22 under drought stress was significantly enriched in the following pathways: biosynthesis of secondary metabolites (9 h: 1093/12 h: 1095), metabolic pathways (9 h: 721/12 h: 737), alpha-linolenic acid metabolism (9 h: 154/12 h: 139), linolenic acid metabolism (9 h: 119/12 h: 53), starch and sucrose metabolism (9 h: 108/12 h: 89), phenylpropanoid biosynthesis (9 h: 86/12 h: 138), plant-pathogen interaction (9 h: 85/12 h: 82), plant hormone signal transduction (9 h: 72/12 h: 50), β-alanine metabolism (9 h: 66/12 h: 53), glycerolipid metabolism (9 h: 60/12 h: 50), glycerophospholipid metabolism (9 h: 54/12 h: 44), MAPK signaling pathway—plant (9h: 49/12h: 51), and circadian rhythm—plant (9 h: 47/12 h: 103) (Figure 8A,B). The biosynthesis of secondary metabolites in stems of FH18 under drought stress was significantly enriched in the following pathways: biosynthesis of secondary metabolites (9 h: 1300/12 h: 1100), metabolic pathways (9 h: 844/12 h: 664), alpha-linolenic acid metabolism (9 h: 89/12 h: 115), starch and sucrose metabolism (9 h: 80/12 h: 61), plant hormone signal transduction (9 h: 72/12 h: 46), and phenylpropanoid biosynthesis (9 h: 67/12 h: 91) (Figure 8C,D).

Based on group comparisons, the differential genes were used for functional enrichment and were involved in various biological processes. The common genes in the comparison group showed that under drought stress, differential genes in peanut leaf tissues were significantly enriched in four pathways: metabolic pathway, biosynthesis of secondary metabolites, carbon metabolism, and phenylpropane biosynthesis. Differential genes in peanut stem tissues were significantly enriched in six pathways: secondary metabolite biosynthesis, metabolic pathway, α-linolenic acid metabolism, starch and sucrose metabolism, plant hormone signal transduction, and phenylpropane biosynthesis.

### 2.7. Analysis of Weighted Gene Co-Expression Network Analyses (WGGNA) Under Drought Stress

In total, 20,982 DEGs were identified using transcriptome sequencing. Dynamic tree cutting generated 21 modules based on expression similarity.

**Figure 9:**
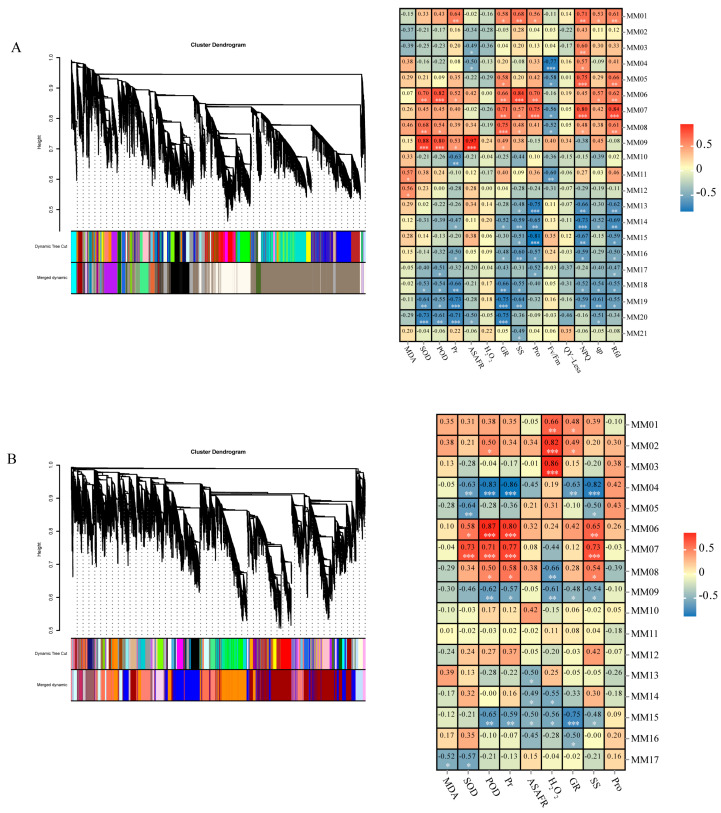
WGGNA of effectively expressed peanut genes under acute drought stress during the seedling stage. (**A**) Hierarchical cluster tree showing the co-expression modules in peanut leaf tissues identified using WGCNA. Each leaf in the tree represents a single gene. Twenty-one modules are labeled with different colors to represent major tree branches, and correlation analysis was performed between gene co-expression network modules and physiological indices. (**B**) Hierarchical cluster tree showing the co-expression modules in peanut stem tissues identified by WGCNA. Each leaf in the tree represents a single gene. Seventeen modules were labeled with different colors to represent major tree branches, and correlation analysis was performed between gene co-expression network modules and physiological indices. For (**A**,**B**), the horizontal axes represent different physiological traits, and the vertical axes represent the eigengenes in each module. Each frame contains the corresponding correlations and *p*-values (* *p* < 0.05, ** *p* < 0.01, *** *p* < 0.001). In the co-expression network, the critical degree of each gene is indicated by the color depth, which indicates a higher critical degree of the gene.

Gene expression is often closely associated with certain traits. Therefore, for leaf tissues, the correlation between the expression of each module and physiology, biochemistry, and chlorophyll fluorescence was analyzed. The MM06 module was positively correlated with physiology and biochemistry, whereas the MM13, MM16, and MM19 modules were negatively correlated with osmotic regulatory substances. Four modules—MM06, MM13, MM16, and MM19—were significantly correlated with specific traits (Figure 9A). For stem tissues, the correlations between module expression, physiology, and biochemistry were analyzed. Modules MM02 and MM06 were positively correlated with physiological and biochemical traits, whereas MM04 was negatively correlated with antioxidant enzyme activity. Three modules—MM02, MM04, and MM06—were significantly correlated with physiological and biochemical traits (Figure 9B).

Correlation analysis was performed on the differentially expressed genes in the seven modules, and the 200 pairs of genes with the highest correlation were selected to construct the co-expression network. Genes at the center of the regulatory network are often called core genes and are key regulators of the expression of upstream and downstream genes. These genes have a higher correlation with other genes than that of non-key genes and play important roles in biological processes.

**Figure 10:**
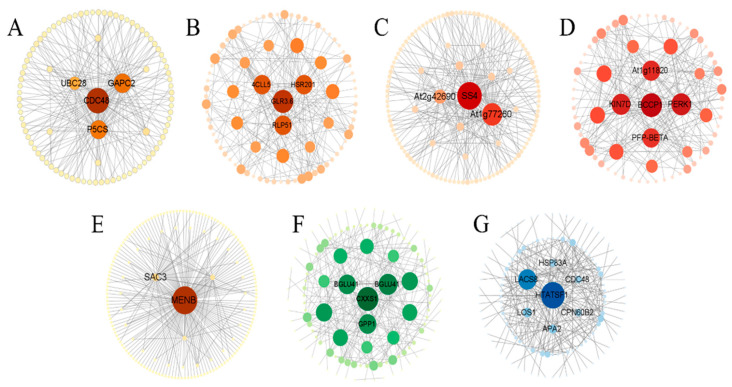
Co-expression networks of key genes in seven peanut modules under acute drought stress during the seedling stage. Co-expression networks of key genes in the leaf tissue module. (**A**) MM06, (**B**) MM13, (**C**) MM16, and (**D**) MM19; co-expression networks of key genes in the stem tissue module: (**E**) MM02, (**F**) MM04, and (**G**) MM06. The size of the nodes indicates the connection degrees of the genes in the network; larger nodes represent a greater number of connections with other genes.

In this study, we focused on four core genes in leaf tissues. The MM06 module contains a gene encoding delta-1-pyrrolin-5-formate synthase isoenzyme X2 (*P5CS*), which catalyzes proline synthesis (Figure 10A) [28]. The MM13 module contained a gene encoding 4-coumarin-CoA ligase-like 5 (*4CLL5*), which is involved in various steps in the linolenic acid metabolic pathway and is key for the synthesis of JA in this pathway (Figure 10B) [29]. The MM16 module contains genes encoding starch synthetase 4 and chloroplast/amyloid isomer X1 (*SS4*), which are involved in the synthesis of starch particles (Figure 10C) [30]. The MM19 module contains a gene encoding plasma membrane corpus callosum binding protein 3 (*At1g11820*), which regulates leaf epidermal growth and development. Controlling epidermal formation using this gene can improve plant tolerance to drought stress (Figure 10D) [31]. Three core genes were studied in the stem tissues. The MM02 module contains a gene (*MENB*) encoding the hypothetical protein KY285_023190, which is involved in photosynthesis (Figure 10E) [32]. The MM04 module contains a gene encoding β-glucosidase 41 isoenzyme X2 (*BGLU41*), which is involved in glucose synthesis (Figure 10F). The MM06 module contains a gene encoding long-chain acyl-CoA synthetase 8 (*LACS8*), which is involved in oil biosynthesis [33] (Figure 10G).

To verify the gene expression data obtained from RNA-Seq, we selected 18 genes involved in flavonoid biosynthesis, peroxisomes, photosynthesis-antenna proteins, and the photosynthetic synthesis pathway under drought stress for qRT-PCR analysis. Under drought stress, RNA-Seq and qRT-PCR showed good agreement with the relative expression levels of all candidate genes, confirming the reliability and accuracy of the RNA-Seq analysis in this study (Supplementary Figures S2 and S3).

### 2.8. Pathway Changes in Peanut Shoots in Response to Drought Stress

**Figure 11:**
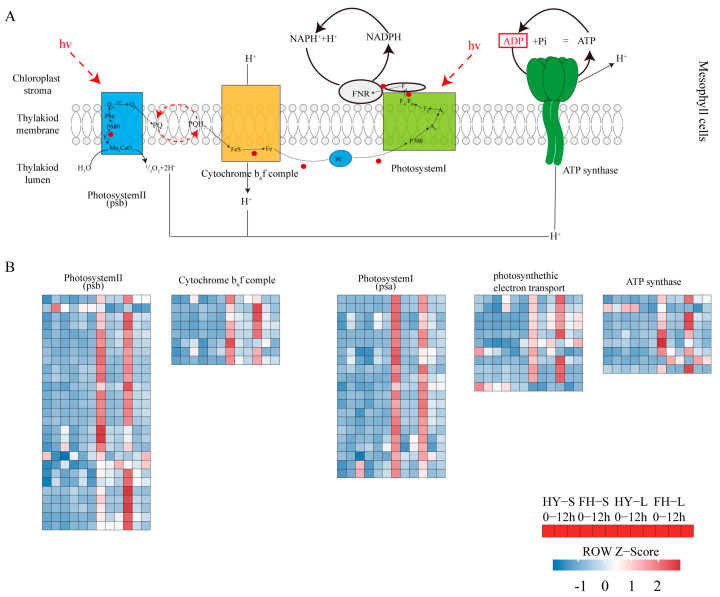
Heatmap showing differentially expressed genes (DEGs) in response to acute drought stress involved in photosynthesis and electron transportation pathways. (**A**) The model of photosynthesis and electron transportation pathways in peanut mesophyll cells. Photosysytem II (psb), cytochrome b_6_f complex, photosystem I, and ATP synthase are anchored in the thylakiod membrane. The oxygen-evolving complex in photosystem II resolves water into two molecules of hydrogen and one molecule of oxygen. Electrons reach plastoquinone (PQ) through a circulatory mechanism, which in turn releases a hydrogen ion in the thylakoid. The cytochrome b_6_f complex transfers electrons from plastoquinone to plastocyanin (PC), which then transfers electrons to PSI. Finally, electrons reach ferredoxin (Fd) and are reduced. As hydrogen ions flow out of the thylakoid membrane due to a concentration gradient, ATP is synthesized by ATP synthase. (**B**) Each color patch on the heatmap represents the FPKM value. Red and blue indicate significant upward and downward expression, respectively (log_2_|fold-change| ≥ 1).

It is pertinent to elucidate the pathways in peanuts affected by drought stress. In particular, photosynthesis is the key to understanding the damage caused by acute drought stress, as the expression of all components related to photosynthesis was downregulated in leaves exposed to drought stress conditions (Figure 11; Table S2). The expression of genes encoding *PSB28*, *PNSL3*, and *PNSL2* in PSII was significantly downregulated in FH18 and less significantly downregulated in HY22, in which *arahy.Tifrunner.gnm2.ann1.T46WBJ* was upregulated after 12 h of the drought treatment. Gene expression related to PSI showed a similar downregulation pattern, but the genes *arahy.Tifrunner.gnm2.ann1.QW0L8Q* and *arahy.Tifrunner.gnm2.ann1.TN6DUA* exhibited upregulation after 12 h, and it was more significant in the leaves of HY22 than that in the FH18. *arahy.Tifrunner.gnm2.ann1.E2679H,* which encodes *PETH* related to photosynthetic electron transport, was upregulated in HY22 cells after 12 h. This suggests that while the photosynthetic system is severely impacted under acute drought stress, the damage is less pronounced in HY22 than that in FH18.

Other plant parts, such as stems, flowers, seeds, spikes, petioles, and roots, can also conduct photosynthesis under stress conditions [34]. However, gene expression related to photosynthesis in the stems of HY22 and FH18 did not change significantly during the drought treatment (Figure 11; Table S2). *arahy.Tifrunner.gnm2.ann1.RVN5Z1*, which encodes *psbW*, was upregulated after 9 h in HY22, whereas it was downregulated in FH18 and in the leaves of both cultivars. Similarly, *arahy.Tifrunner.gnm2.ann1.0W708Z*, which encodes *psbB*, was upregulated in the stems of both cultivars, with significant expression in HY22 after 12 h, whereas it was downregulated in the leaves of both HY22 and FH18. Similarly, *arahy.Tifrunner.gnm2.ann1.UAEM0K* and *arahy.Tifrunner.gnm2.ann1.3I3T69*, which are related to PSI, were significantly upregulated in the stem of HY22 but downregulated in the leaves of both cultivars. *arahy.Tifrunner.gnm2.ann1.AR01JJ*, *arahy.Tifrunner.gnm2.ann1.ZQ3F6U*, *arahy.Tifrunner.gnm2.ann1.X4SEKR*, and *arahy.Tifrunner.gnm2.ann1.X5CP5W*, which encode pea plastocyanin gene (PETE) in HY22 after 9 h, were upregulated in stem and downregulated in FH18, and the same downregulation was observed in the leaves of the two cultivars. Three genes, namely *arahy.Tifrunner.gnm2.ann1.39S8LV*, *arahy.Tifrunner.gnm2.ann1.F0UT86*, and *arahy.Tifrunner.gnm2.ann1.NN0MZU*, which encode components of the cytochrome b6f complex, maintained gene expression at 9 h and were downregulated after 12 h in HY22, whereas they were downregulated as drought stress started at 9 h in FH18 and in the leaves of the two cultivars. One gene, *arahy.Tifrunner.gnm2.ann1.CW3FX9*, encoding *SEND33* of ATP synthase, was downregulated less significantly in HY22 than in FH18, whereas it was significantly downregulated in the leaves of both varieties (Figure 11; Table S2). It was inferred that the stem also had a photosynthetic function during the seedling stage, and it worked differently from the leaves. In addition, numerous genes maintained their function under drought stress in HY22.

**Figure 12:**
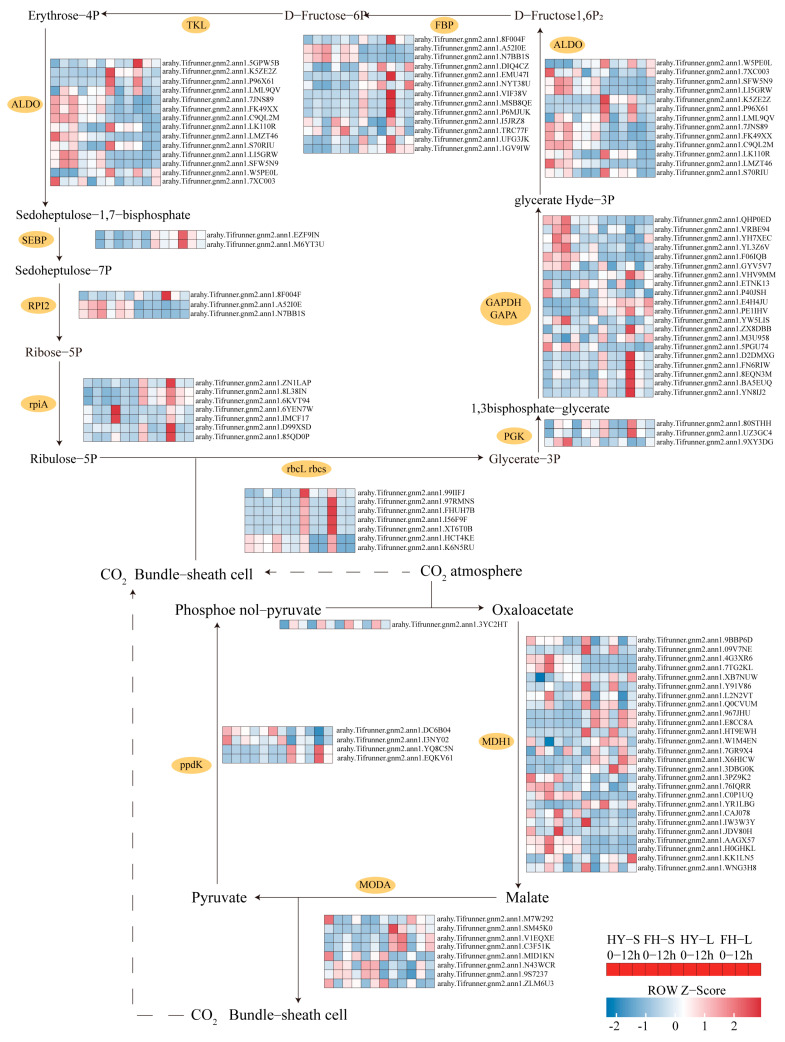
Heatmap showing differentially expressed genes (DEGs) in response to drought stress involved in photosynthetic carbon fixation and the CAM, sucrose, and starch synthesis pathways. Each color patch represents the log_2_ FC value. Red and blue indicate significant upward and downward revisions for each gene (log_2_|fold-change| ≥ 1).

Most genes involved in carbon fixation were downregulated in the leaves of both cultivars. However, the extent of downregulation for ribulose-bisphosphate carboxylase small chain (rbcS) was less severe in the stems of both cultivars, and the downregulation fold was low in the stems of HY22, specifically for *arahy.Tifrunner.gnm2.ann1.FHUH7B*, *arahy.Tifrunner.gnm2.ann1.I56F9F*, and *arahy.Tifrunner.gnm2.ann1.HCT4KE*. Conversely, the genes associated with ribulose-bisphosphate carboxylase large chain (rbcL), such as *arahy.Tifrunner.gnm2.ann1.99IIFJ*, were upregulated in HY22 stems. Similarly, genes encoding phosphoglycerate kinase (PGK3) were upregulated in the stems of both cultivars, although their expression varied in the leaves with *arahy.Tifrunner.gnm2.ann1.YW5LIS* and *arahy.Tifrunner.gnm2.ann1.9XY3DG* being upregulated in HY22 after 9 and 12 h, respectively (Figure 12; Table S2). Furthermore, genes related to glyceraldehyde-3-phosphate dehydrogenase (NADP^+^) (phosphorylating) (GAPA), specifically *arahy.Tifrunner.gnm2.ann1.D2DMXG* and *arahy.Tifrunner.gnm2.ann1.FN6RIW* were upregulated at 9 h and downregulated at 12 h in HY22 stems but remained higher than the levels at 0 h. Conversely, they were downregulated at 12 h in the stems of FH18, showing lower levels than at 0 h. *arahy.Tifrunner.gnm2.ann1.VRBE94* and *arahy.Tifrunner.gnm2.ann1.YL3Z6V* were significantly upregulated in the stems of HY22. Additionally, genes related to fructose 1,6-bisphosphate aldolase (ALDO), including *arahy.Tifrunner.gnm2.ann1.P96X61*, *arahy.Tifrunner.gnm2.ann1.LI5GRW*, and *arahy.Tifrunner.gnm2.ann1.LML9QV* displayed similar upregulation trends in the stems of HY22 (Figure 12; Table S2). Genes encoding fructose-1, 6-bisphosphatase I (FBP), such as *arahy.Tifrunner.gnm2.ann1.P6MJUK* and *arahy.Tifrunner.gnm2.ann1.MSB8QE* were upregulated in HY22 stems compared with FH18 plants but showed less downregulation in HY22 leaves compared to FH18 plants. Four genes encoding tyrosine kinase-like (TKL) proteins were also significantly upregulated in HY22 stems and less so in HY22 leaves. Similar expression patterns were observed for genes related to RPl2 (Figure 12; Table S2).

**Figure 13:**
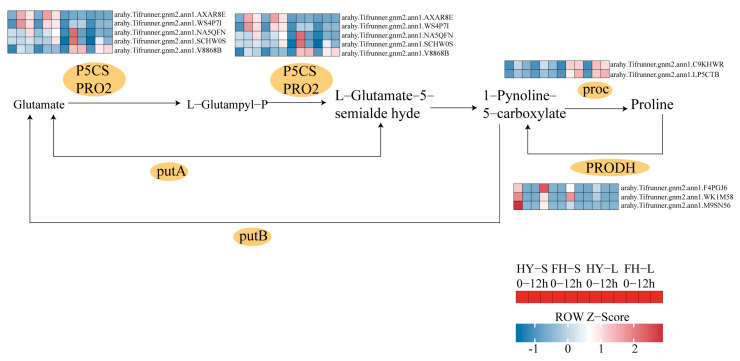
Heatmap showing differentially expressed genes (DEGs) in response to acute drought stress involved in the proline synthesis pathway. Each color patch represents the log_2_ FC value. Red and blue indicate significant upward and downward revisions of each gene (log_2_|fold-change| ≥ 1).

Under drought stress conditions, four genes encoding Δ^1^-pyrroline-5-carboxylate synthase (P5CS)—*arahy.Tifrunner.gnm2.ann1.1W477Y*, *arahy.Tifrunner.gnm2.ann1.AXAR8E*, *arahy.Tifrunner.gnm2.ann1.V8868B*, and *arahy.Tifrunner.gnm2.ann1.WS4P7I*—were significantly upregulated in HY22 shoots. Furthermore, two proC-encoding genes that catalyze the conversion of 1-pyrroline-5-carboxylate to proline, namely *arahy.Tifrunner.gnm2.ann1.77YH77* and *arahy.Tifrunner.gnm2.ann1.0II3I*, were continuously upregulated in the leaves of HY22 under prolonged drought stress (Figure 13; Table S2). Conversely, three genes related to the proline dehydrogenase (PRODH) catalysis—*arahy.Tifrunner.gnm2.ann1.WK1M58*, *arahy.Tifrunner.gnm2.ann1.F4PGJ6,* and *arahy.Tifrunner.gnm2.ann1.M9SN56*—were significantly downregulated in HY22, whereas they were upregulated in FH18 (Figure 13; Table S2). As such, we infer that drought-stressed cultivars could regulate proline synthesis by upregulating the levels of the corresponding genes.

**Figure 14:**
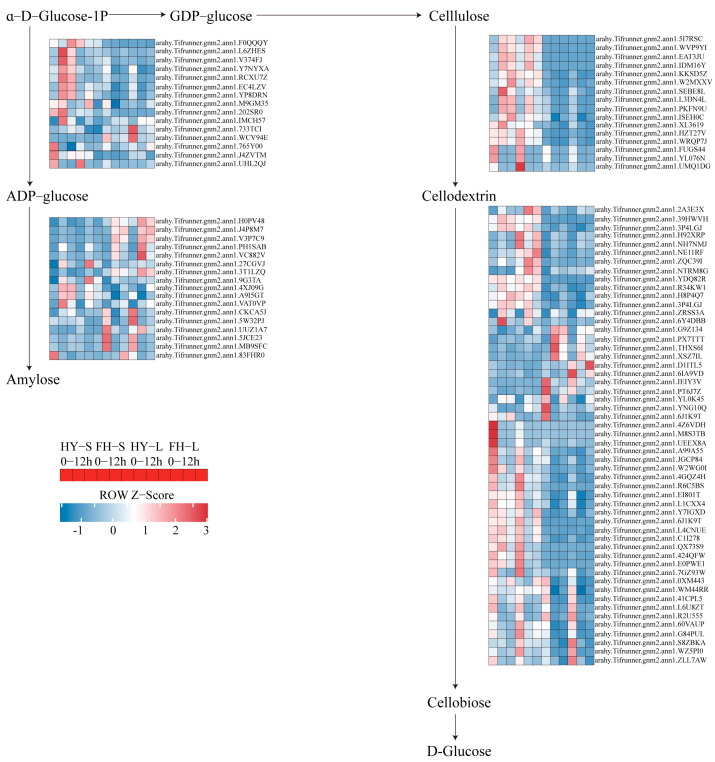
Heatmap showing differentially expressed genes (DEGs) in response to acute drought stress involved in sucrose and starch synthesis pathways. Each color patch represents the log_2_ FC value. Red and blue indicate significant upward and downward revisions of each gene (log_2_|fold-change| ≥ 1).

In the starch and sucrose metabolism pathway, four genes encoding B-glucosidase (CELB), which catalyzes cellulose to cellodextrin, namely *arahy.Tifrunner.gnm2.ann1.FUGS44*, *arahy.Tifrunner.gnm2.ann1.KKSD5Z*, *arahy.Tifrunner.gnm2.ann1.UMQ1DG*, and *arahy.Tifrunner.gnm2.ann1.YL076N*, were initially downregulated at 9 h but slightly upregulated at 12 h in both HY22 and FH18. However, *arahy.Tifrunner.gnm2.ann1.KKSD5Z* in the stem of HY22 exhibited continuous upregulation. Similarly, the genes encoding bglX and bglB followed this pattern of downregulation at 9 h and slight upregulation at 12 h in both cultivars (Figure 14; Table S2). However, some genes were upregulated at 12 h with almost the same expression levels as the control in both cultivars. In contrast, abundant genes encoding ADP-glucose pyrophosphorylase (glgC), glycogen synthases (glgA), and WAXY, which promotes the conversion of α-D-glucose-1P to amylose, were significantly upregulated at 9 h and slightly downregulated at 12 h in both cultivars. Upregulated expression levels at 9 h were more significant in HY22 stems (Figure 14; Table S2).

**Figure 15:**
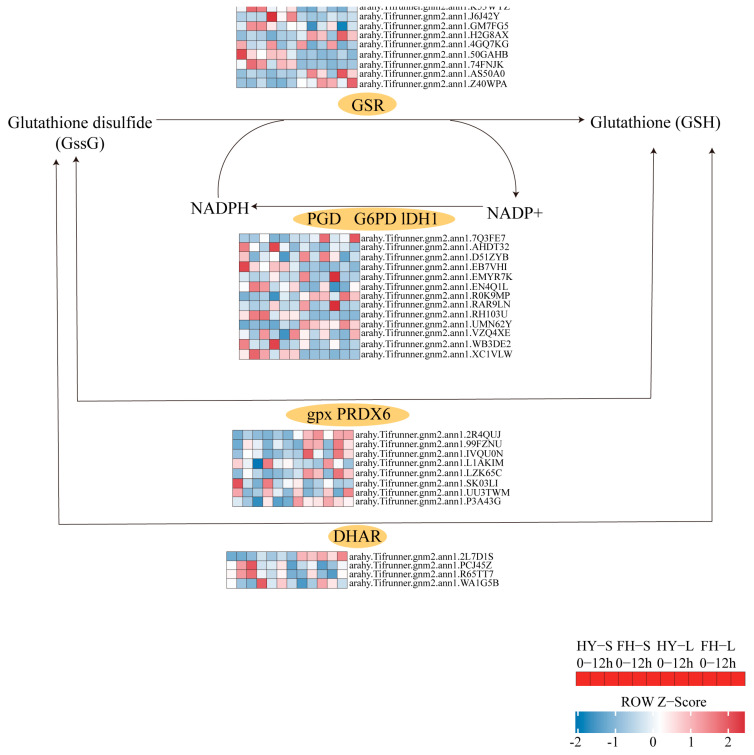
Heatmap showing differentially expressed genes (DEGs) in response to acute drought stress involved in the glutathione pathway. Each color patch represents the log_2_ FC value. Red and blue indicate significant upward and downward revisions of each gene (log_2_|fold-change| ≥ 1).

Most genes encoding glutathione reductase (GSR), glutathione peroxidase (GPX), and glucose-6-phosphate dehydrogenase (G6PD) were upregulated at 9 h but downregulated at 12 h in both HY22 and FH18. However, three genes encoding dehydroascorbate reductase (DHAR) were upregulated at both 9 and 12 h, with *arahy.Tifrunner.gnm2.ann1.R65TT7* and *arahy.Tifrunner.gnm2.ann1.WA1G5B* showing significant upregulation in both the stems and leaves of HY22 (Figure 15; Table S2). Two genes related to NADP-dependent isocitrate dehydrogenase (ICDH) were downregulated at 9 and 12 h in the two cultivars; however, *arahy.Tifrunner.gnm2.ann1.D51ZYB*, in the leaves of HY22, was upregulated compared with the expression in the CK at 12 h. Furthermore, *arahy.Tifrunner.gnm2.ann1.RH103U* exhibited different expression trends in stems and leaves, with constant upregulation in stems and downregulation in leaves of both cultivars (Figure 15; Table S2).

## 3. Discussion

### 3.1. Effects of Drought Stress on the Morphological Characteristics and Water Content of Peanut

Plant leaves are exposed to aerial environments, and their structural characteristics reflect their water-use capacity [35]. The drought responses of leaves primarily improve their water-holding capacity and water-use efficiency, thereby helping plants maintain normal life activities. Under drought conditions, leaf yellowing, curling, and wilting occur, effectively reducing plant transpiration and water loss. Numerous studies have shown that drought stress causes the stem to become thinner, thereby reducing its ability to absorb and transport nutrients. Liu et al. [36] investigated drought resistance among 80 peanut germplasms using leaf morphology as a marker. They found that under drought stress, plants exhibited dwarfed and stunted growth. In this study, we found that drought stress affected shoot morphology in both cultivars to varying degrees. Compared with HY22, FH18 showed more obvious phenotypic changes and was more sensitive to damage after 24 h of drought treatment (Figure 1A).

Water content is a crucial factor in plant health and directly affects plant photosynthetic capacity [37]. Under drought conditions, reduced water loss from the plant body indicates stronger water retention ability. This also indicates that plants can efficiently meet the needs of their life activities with only limited water resources. Akter [38] found that the water content of wheat leaves decreased under drought stress, and this decrease was more obvious as the drought stress increased. Similarly, the aboveground water content of the two peanut cultivars used in this study decreased due to drought stress. With an increasing water deficit, the water loss rates of the two peanut cultivars accelerated, particularly in L3 and S2, the upper morphology terminals of the plant, which were more prone to water loss. This suggests that the plants were unable to maintain the cell turgor necessary for expansion, resulting in stunted growth or even death (Figure 1). The water loss rate of the drought-sensitive cultivar FH18 was significantly higher than that of the drought-resistant cultivar HY22 (Figure 1). Meher et al. [39] found that under severe drought stress, peanuts lose their ability to sustain water content. This could be used as a parameter for selecting peanut genotypes with better cell turgor maintenance.

### 3.2. Effects of Drought Stress on Shoot Physiology, Biochemistry, and Regulation Mechanisms

The generation and removal of ROS in plants can create a dynamic equilibrium state. In response to various abiotic and biotic factors, plants accumulate ROS; however, ROS accumulation can exceed physiological limits and cause damage to biological macromolecules [40]. When drought stress occurs, abnormal ROS metabolism is significantly induced, leading to excessive ROS accumulation and cell membrane damage [41]. MDA is the final product of membrane lipid peroxidation and reflects the degree of plant damage under stress conditions. MDA content in peanut leaves first increases and then decreases under drought stress [42]. In the present study, we found a significant vertex at 12 h in the shoots of FH18 (Figure 2A), indicating that drought-sensitive cultivars have undergone cell membrane damage during the early stage of drought or even under mild drought stress [39]. ROS is effectively removed by antioxidant enzymes, such as SOD, POD, and GR, to maintain ROS balance in the body and relieve drought damage [43]. SOD primarily catalyzes the disproportionation of superoxide anion radicals to generate oxygen and hydrogen peroxide and eliminates the toxic effect of free radicals under the synergistic effect of POD [44]. GR maintains a high GSH/oxidized glutathione (GSSG) ratio, thereby protecting cells from oxidative damage, and usually functions as a complementary enzyme in the ascorbate-glutathione cycle [45,46] (Figure 3). There are two genes encoding GRs: GR1, which encodes a cytosolic protein, and GR2, which encodes a dual-targeted chloroplast and mitochondrial GR with a functional role in plant development [47]. However, GR1 is not necessary for plant development and cannot compensate for the shortage of glutathione in organelles [46,48]. In this study, only GR1 was detected, and further studies will be required to investigate GR2 (Figure 15; Table S2). Although SOD activity occurred significantly earlier in FH18 at 16 h than in HY22, POD and GR activities were significantly higher in HY22 shoots, even after 20 h, indicating that drought-resistant cultivars could eliminate ROS under drought stress (Figure 3).

In addition, drought stress reduces cell dilation, resulting in osmotic stress. Osmoregulatory substances, such as free proline, soluble sugar, and soluble proteins, accumulate in plants to regulate osmotic stress [49]. Proline provides sufficient free water for plants, preventing excessive water loss from affecting normal plant life activities [50]. Under drought stress, four genes encoding P5CS were significantly upregulated in the shoots of HY22, and two genes encoding P5CR, which catalyze the conversion of L-pyrroline-5-carboxylate to proline, were continuously upregulated in HY22 under prolonged drought stress (Figure 13; Table S2). We inferred that the drought stress cultivar could regulate proline synthesis by upregulating the expression of the corresponding genes (Figure 4C,F). Deng et al. [51] found that under drought stress, plants with silenced *P5CS* exhibited Pro degradation and decreased P5CS gene expression. Similarly, with prolonged drought treatment, the expression of *P5CS* increased, and the Pro content was enhanced [52]. P5CS and P5CR are the key rate-limiting enzymes that catalyze the conversion of glutamate to proline; P5CS responds to dehydration but also directly participates in plant growth and development and is involved in embryo abortion at the late stage of seed development; consequently, it is called a housekeeping gene [53,54,55]. Furthermore, NADPH is used in proline biosynthesis as an electron donor, and NADP^+^ is then used as an electron acceptor in PSI, thereby decreasing singlet oxygen production. This further indicates that proline helps sustain the required NADPH/NADP^+^ ratio [23,56]. Notably, the gene related to PROH, which catalyzes proline to 1-pyrroline-5-carboxylate, was significantly downregulated in HY22 after drought treatment, whereas an opposite response was observed in FH18 (Figure 13; Table S2). Under drought stress, proline is also controlled by the catabolic genes *PDH* and *P5CDH* and normally exhibits downregulation; however, in some cases, proline is upregulated with the progression of drought [57,58,59]. These results indicate that dynamic changes in proline content during dehydration play vital roles in plant survival. Therefore, proline accumulation is an effective parameter for selecting drought-resistant genotypes [60].

Similarly, soluble sugars (sucrose, glucose, and fructose) are significantly increased and can provide osmotic adjustments under drought stress because they are involved in maintaining plant structure and growth [61]. In this study, the soluble sugar content increased steadily at 12 and 24 h in the leaves and stems of HY22 and FH18 (Figure 4B,E). Xu et al. [62] found that soluble sugar content increased significantly in the leaves and roots of susceptible rice plants but decreased in the stems of tolerant and susceptible rice. Similarly, it decreased in drought-tolerant, drought-sensitive, and fast-recovery rice cultivars under drought stress [63]. In the starch hydrolysis pathway, genes related to *bglX*, *bglB*, and *CELB* were primarily downregulated in leaves but were first elevated and then downregulated in the stems of the two cultivars. However, one gene, *arahy.Tifrunner.gnm2.ann1.KKSD5Z*, related to *CELB*, was consistently upregulated in the stems of HY22, which may explain why the soluble sugar content was higher at 12 h in the stems of HY22 (Figure 14; Table S2). In the amylose synthesis pathway, *arahy.Tifrunner.gnm2.ann1.L6ZHES* and *arahy.Tifrunner.gnm2.ann1.V374FJ* were significantly elevated at 9 h in the stems of HY22, indicating that the amylose accumulation increased in HY22, thereby storing energy to resist stress (Figure 14; Table S2). Soluble sugars play complex roles in plant growth regulation, such as in photosynthesis, carbon partitioning, and carbohydrate and lipid metabolism [63,64]. Soluble proteins, which are hydrophilic, tend to accumulate autonomously during drought stress conditions, enhancing the water retention capacity of cells (Figure 4A,D). Initially, under drought stress, the SP content in Pennisetum Sinese Roxb seedlings increases, aiding in maintaining a low osmotic potential in the cells and preventing dehydration. However, as drought stress prolongs, the plant’s drought resistance mechanisms gradually “collapse,” leading to a decrease in SP and consequent severe damage to the plants [65]. The results of this study showed that changes in osmoregulatory substances in the leaves and stems of the two varieties were similar; they first increased and then decreased.

The results show that HY22 has better drought resistance mechanisms than FH18, and it can effectively activate its defense mechanisms under drought stress and greatly reduce the potential damage. The peak responses of antioxidant enzymes and osmoregulatory substances in the stem tissues typically occurred earlier than those in the leaves, largely because the distance between the stems is close to the root systems, allowing them to respond more quickly to drought stress and activate defense mechanisms swiftly (Figure 3 and Figure 4). In HY22, a drought-resistant cultivar, the peak levels of these substances in the leaves usually occurred at 24 h, whereas in FH18, a more drought-sensitive cultivar, they peaked at 16 h. Similarly, the peaks in the stems of HY22 were at 12 h, compared to 9 h in FH18. Under drought stress, the levels of antioxidant enzymes and osmoregulatory substances in HY22 were significantly higher than those in FH18. This suggests that drought-resistant cultivars possess more stable and enduring drought response mechanisms.

### 3.3. Effects of Drought Stress on Peanut Shoot Anatomy Structures

The anatomical structures of an aboveground plant are vital for its physiological characteristics, especially its response mechanisms to water deficiency. As the soil moisture decreases, plants experience decreased xylem pressure, which leads to stomatal closure, reduces hydraulic conductivity, and increases drought-induced mortality [66,67,68]. Xylem provides the most energy-efficient means of transporting water through the stems and leaves, but it is also the site of the most significant hydraulic conductivity losses during drought. Qaderi et al. [68] reported that drought decreased the density of xylem vessels and tracheids. The results of this study showed that the stem structure of FH18 was seriously damaged after drought stress; the epidermal layer was wrinkled, the boundary between the epidermis and cambium was damaged, the vascular bundle sheath became blurred, and the pith appeared “hollow” (Figure 5B). Additionally, we tested those two cultivars in a rain shelter with drought and without drought treatment, for which the water control began at the three-leaf stage, and the similar cell structure appeared at 9 days and 15 days (Figure S4). These characteristics may be the result of programmed cell death or non-programmed cell death. Strengthening the water duct arrangement to improve the ability of plants to absorb water from the soil is an effective way of preventing plant atrophy when soil water is not replenished. In summary, the drought resistance mechanisms of stems include water storage, water retention, enhanced water transport, and protection after water loss [15]. In contrast, HY22 exhibited less damage, a relatively complete organizational structure, and maintained the functions of transporting nutrients and water (Figure 5B), particularly in the vascular cambium during drought conditions. The initiation and differentiation of the vascular cambium are pivotal steps in xylem development, and they are affected by humidity, temperature, and rainfall [69,70]. Patel et al. [70] found that the cambial activity of plants is very sensitive to drought, and a shortage of soil water results in delayed cell division in the vascular cambium. As a pivotal station between the leaf and stem, the petiole plays an important role in transporting water, nutrients, and assimilating substances. The structure of the petiole was similar to that of the stem, as were the structural changes under drought stress. Compared with HY22, the petiole structure of FH18 was seriously damaged, the epidermis was depressed, the vascular bundle sheath edge structure was blurred, the vascular bundle sheath and xylem edge were disrupted, and a large number of cells in the pith were disrupted (Figure 5C), indicating that drought suppressed cambial cell division and inhibited cell enlargement by reducing turgor pressure [71]. It is important to identify the correlation between the particular structural characteristics of vascular development and the efficiency of water transport in peanuts. Notably, drought-resistant cultivars sustained the anatomical structures of the stems and petioles to facilitate the transport of water, nutrients, and essential substances for plant growth and development under drought stress (Figure 5B,C and Figure S4B,C). There was an increase in the osmotic potential, that is, soluble sugar, protein, and proline in the xylem sap obtained from HY22, and this was sustained under prolonged drought stress (Figure 5 and Figure S4), which helped to maintain a normal water flow. Similar results have been reported in other species, such as grapevines (*Vitis vinifera* L.) [71].

Drought stress affects xylem pressure, leading to stomatal closure. In this study, stomatal closure was observed using a fluorescence microscope in HY22 after 12 h of drought stress, whereas it was observed in FH18 after 16 h (Figure S5). To adapt to drought stress, the leaf thickness, palisade thickness, and spongy thickness are significantly modified to reduce water loss [72]. Specifically, the thickness of the palisade tissue increases, that of the sponge tissue decreases, the intercellular arrangement becomes denser, and the shape changes from slender to short [5,73]. However, the leaf structures of different peanut varieties respond differently, and the leaf thickness, palisade tissue thickness, and palisade tissue/sponge tissue thickness ratio are higher in drought-resistant varieties [74]. Compared with the control, the thickness of the leaf and spongy layers decreased, the thickness of the palisade and palisade/spongy layer increased, and the leaf cells were loosely arranged and shortened after drought stress (Figure 5A; Table 1). The leaf thickness of HY22 decreased less than that of FH18, the thickness of the palisade/sponge increased more than that of FH18, and the change in cell shape was less pronounced than that of FH18. Thus, HY22 could effectively reduce plant damage by adapting the leaf structure to better drought stress conditions (Figure 5A and Figure S4A).

### 3.4. Effects of Drought Stress on Peanut Photosynthetic Regulation Mechanisms

Abiotic stress conditions can change the photosynthetic physiology of plant leaves. The chlorophyll fluorescence parameter is an internal index that links plant photosynthesis and the environment, reflects the physiological status and mechanism of plant photosynthesis, and is widely used to study plant stress [75]. Under drought stress, chlorophyll fluorescence parameters of winter wheat, such as Fv/Fm, decrease, resulting in a reduced photosynthetic rate. In addition, NPQ showed an increasing trend, and the PS II reaction center of cotton leaves enhanced their ability to dissipate excess light energy; most of the absorbed light energy was used for heat dissipation to protect the PS II reaction system from photo suppression [76]. Rfd reflects the potential photosynthetic quantum conversion efficiency of leaves and their photosynthetic activity [77]. In this study, under drought stress conditions, the Fv/Fm and Rfd of peanuts decreased, but NPQ increased. Compared with HY22, the NPQ in FH18 increased more significantly, indicating that the photosynthetic system in FH18 was more severely destroyed under drought stress (Figure 6). When these two cultivars were planted under rainfall shield conditions, NPQ was still significantly higher in FH18 than in HY22 after 9 and 15 d of the drought stress conditions (Figure 6 and Figure S6). However, 9 h after drought stress, Rfd was significantly lower in HY22 than in FH18, but it increased significantly after 12 h in HY22 and surpassed that of the FH18. It was inferred that with prolonged drought stress, the PS II reaction center of drought-resistant cultivars was less damaged, and the photosynthetic rate was sustained (Figure 6 and Figure S6). In addition, the gene expression encoding PNSL2 and PNSL3 was less significantly downregulated in leaves from HY22 than that in those from FH18, except for the upregulation of *arahy.Tifrunner.gnm2.ann1.T46WBJ* after 12 h in HY22 (Figure 11; Table S2). PNSL2 and PNSL3 belong to the PsbQ homolog and are key factors in the stability of the lumenal subcomplex, whereas PsbQ is vital for PSII stability because it cooperates with PsbP to disassemble and/or reassemble PSII complexes and smooth the PSII repair cycle [78,79,80,81]. *arahy.Tifrunner.gnm2.ann1.72UZ71* and *arahy.Tifrunner.gnm2.ann1.FRV526*, which encode Psb28, were less altered in HY22 than in FH18 (Figure 11; Table S2). Psb28, in chloroplasts, is a 13 kDa SP, which was first identified as a cofactor related to the biogenesis of PSII and maintenance of PSII activity under abiotic stress. Furthermore, it is reportedly involved in the stabilization of a/b-binding (CAB)-like proteins (SCPs) and CP47 of PSII under stress conditions, and its two forms, Psb28–RC47 and Psb28–PSII, protect PSII from photodamage during assembly [82]. Similarly, the decreased rates of Fv/Fm, Rfd, and other parameters for sugarcane during the seedling stage were correlated with prolonged drought treatment, indicating that the primary light energy conversion efficiency and potential photosynthesis capacity of photosystem II decreased but also reflected the resistance ability of crops to drought stress [77].

Intriguingly, changes in the expression of genes related to photosynthesis in the stems were also observed. Green plants, as sedentary life, are equipped with brilliant photoautotroph systems, and the leaf, as a primary organ for photosynthesis, has been well studied; however, other plant parts have also participated in this process, such as stems, panicles, flowers, seeds, and even roots, when exposed to light [83]. These parts use the carbon dioxide produced by the plant during respiration, which is stressed under abiotic and biotic conditions, thereby significantly increasing the yield and possibility of subsistence [34]. However, the chloroplast structures and functions remain unclear. In this study, some of the genes related to PSII, PSI, cytochrome b6f complex, ATP synthase, and photosynthetic electron transport were upregulated after 9 h and downregulated after 12 h in HY22, but downregulated in FH18 and downregulated in the leaves of both cultivars (Figure 11; Table S2). Interestingly, gene expression regulation was different in the stems, and it was inferred that different response mechanisms existed between the leaves and stems. Photosynthesis in the non-leaf part consisted of the C3, C4, and crassulacean acid metabolism (CAM) pathways. The C3 pathway is common in both leaf and non-leaf tissues. The C4 pathway has been identified in the stems and petioles of tobacco and celery, in wheat and barley seeds, and in cucumber [84,85,86,87,88]. It has also been observed in the epidermal and mesophyll cells of *Ottelia* leaves with non-Kranz NAD-ME subtype characteristics [89]. Although there is no evidence directly supporting CAM in non-leaf tissues, it supports large leaf plants to avoid quick water loss under arid conditions [34]. Spontaneously, there were many stem gene expression changes related to CAM in the two peanut cultivars (Figure 12; Table S2), indicating that under drought stress, non-leaf tissues adopted the CAM pathway to avoid water loss. It was found that the stem, leaf sheath, and spike contributed to an increase in grain weight by almost 40% due to photosynthesis under stress conditions [90,91]. This leads to the idea of identifying chloroplast characteristics of the non-leaf photosynthetic parts in this ongoing study, especially under adverse conditions [92].

There are three stages in the Calvin cycle: carbon fixation (carboxylation), reduction, and regeneration. Initially, the carboxylation rate mainly determines the rate of carbon assimilation, which is catalyzed by ribulose 1, 5-bisphosphate carboxylase/oxygenase (Rubisco) (rbcL rbcs). Noticeably, the genes encoding rbcS were dramatically downregulated in the leaves of both cultivars, whereas it was less significant in the stems, and one gene encoding rbcL was significantly elevated in the stem of HY22 but downregulated in FH18 and in the leaves of both cultivars. This suggests that under drought stress, the carboxylation rate was significantly inhibited in the photosynthetic position; however, in the drought-resistant cultivar, the stem may play a compensatory role. Furthermore, in the reduction stage, the gene encoding PGK was mostly upregulated at 9 h and downregulated at 12 h, but was still higher than at 0 h in the stem of HY22, whereas it was upregulated in the leaves of the two cultivars (Figure 12; Table S2). It has been reported that PGK is involved in producing ATP in glycolysis, except for producing 1 of 3-bisphosphoglycerate in the Calvin cycle, which is performed by two types of PGK isozymes: cytosolic and chloroplast PGK [93]. These two isozymes interact to produce fatty acid substrates and are involved in carbon partitioning [94]. Joshi et al. [95] found that over-expressing *OsPGK2a-P* in plants increased water flux by increasing stomatal density and had more efficient photosynthesis under salt stress. Specifically, there was also significantly upregulated expression of GAPA in the stem of HY22, a subunit of chloroplast GAPDH [96]. ALDO, sedoheptulose 1,7-bisphosphatase (SBP), and transketolase (TK) are involved in the Calvin cycle and positively related to photosynthesis [97]. ALDO, FBP, and SBP can control the carbon flux and are constantly upregulated in the stems of HY22 but downregulated in the leaves of both cultivars (Figure 12; Table S2) [97,98]. Uematsu et al. [99] found that *ALDO* overexpression in tobacco promoted ribulose 1,5-bisphosphate regeneration and CO_2_ fixation. As the first catalyzing enzymes involved in irreversible reactions, FBP and SBP facilitate the conversion of triose phosphates to sucrose to increase photosynthesis capacity and carbohydrate accumulation [100]. Gong et al. [101] introduced *FBP/SBPase* into rice and found that the carboxylation efficiency and net photosynthetic rate significantly changed. In this study, the genes in the HY22 stems were also upregulated, which confirmed that under drought stress, the photosynthetic functions in the leaves of the two cultivars were damaged; however, in the drought-resistant cultivar, the stem served as a substitution function to improve plant survival.

## 4. Materials and Methods

### 4.1. Materials and Experimental Design

The experiments were performed at the Peanut Institute of Shenyang Agricultural University, China, from 2021 to 2023. Cultivars Huayu 22 (HY22) and Fuhua 18 (FH18) were selected based on their drought-resistant and drought-sensitive characteristics, respectively, as identified in our previous study [25]. Robust seeds were selected, soaked in a Petri dish for approximately 5 h at 20–25 °C, and then germinated. When the radicles measured approximately 1 cm, one seedling was planted in each pot (12.4 × 16.4 cm) filled with wet vermiculite. The seedlings were then transferred to a culture chamber set at 28 °C/23 °C (day/night) with a 16/8 h light/dark period, 60% humidity, and a light intensity of 500 μmol/m^2^/s.

When the seedlings reached the three-leaf stage, featuring one central growth axis (3rd true leaf unfolded), they were transferred into a hydroponic box containing 6000 mL of culture solution for 6 h, followed by a transfer to a 20% PEG-6000 solution [25]. Taking 0 h as control, the experimental plants underwent drought treatment of 3, 6, 9, 12, 16, 20, 24, and 36 h to evaluate their morphological, physiological, biochemical, and chlorophyll fluorescence characteristics. Leaf and stem samples were selected for paraffin sectioning and transcriptome sequencing at 0, 9, and 12 h. For sampling, leaves and stems were divided into three and two layers, respectively, to elucidate spatiotemporal differences in their responses to drought stress; The layers were labeled from root to apical point as follows: leaf 1 (L1), leaf 2 (L2), and leaf 3 (L3); and stem 1 (S1) and stem (S2) (Supplementary Figure S7).

### 4.2. Drought-Related Indicators

Morphological changes in peanut plants under drought stress were recorded using a digital camera (Nikon D800, Tokyo, Japan). Three plants with similar growth during each period were selected, and the fresh weights of their leaves and stems were measured. These leaves and stems were then dried in an oven at 85 °C until a constant dry weight was achieved.

Leaf and stem samples from the peanuts (0.2 g) were collected and ground into a powder using a high-throughput tissue grinder (SCIENTZ-192, Hangzhou, China). Subsequently, 2 mL of pre-cooled PBS phosphate buffer was added, and the mixture was placed in a VETEX SHAKER QL-866 until thoroughly mixed. The mixture was then centrifuged (HEMA TGL-16R, Hangzhou, China) at 13,000 rpm for 15 min, and the supernatant, containing the crude enzyme liquid, was collected. It was used to determine the levels of malondialdehyde (MDA) (thiobarbituric acid method), SOD (NBT method), POD (guaiacol oxidation method), and SP (Coomasi brilliant blue method), as described by Wang et al. [25]. Additionally, 0.5 g of the peanut leaves and stem tissues were sampled for analysis of SS (indanin colorimetric method) and Pro (phenol method) as used by Wang et al. [25]. Anti-superoxide anion radical (ASAFR), H_2_O_2_, and glutathione reductase (GR) levels were determined using kits (A052-1-1, A064-1-1, and A062-1-1) from the Nanjing Jiancheng Bioengineering Institute, China, following the established protocols.

The tissue structures of the leaves and stems were observed using the paraffin section method [102]. Functional leaves from three peanut plants with identical growth statuses were selected to observe stomatal opening, determine chlorophyll fluorescence parameters, and collect chlorophyll fluorescence images using FluorCam Photon system instruments (FC800-313, Drásov, Czech Republic).

### 4.3. Transcript Sequencing of Drought-Stressed Plant Parts

#### 4.3.1. RNA Extraction, cDNA Library Construction, and Sequencing

Three biological replicates were selected, each comprising three technical replicates. H-0-L, H-9-L, and H-12-L represent leaf tissue samples of HY22 after 0, 9, and 12 h of drought stress, respectively. Similarly, H-0-S, H-9-S, and H-12-S denote stem tissue samples of HY22 at the same time intervals. Total RNA was extracted using TRIzol kits (Invitrogen, Carlsbad, CA, USA) following standard protocols. RNA quality was assessed using an Agilent 2100 Bioanalyzer (Agilent Technologies, Palo Alto, CA, USA) and verified using RNase-free agarose gel electrophoresis. After extraction, rRNA was removed using a Ribo-Zero^TM^ magnetic kit, and prokaryotic mRNA was enriched (Epicenter, Madison, WI, USA). The enriched mRNA was fragmented using a crushing buffer, and cDNA was inverted using the NEBNext Ultra RNA Library Prep Kit for Illumina (NEB #7530, New England Biolabs, Ipswich, MA, USA). The purified double-stranded cDNA fragments were end-repaired, a single base was added, and an Illumina sequencing adapter was attached. The linking reaction was purified using AMPure XP beads (1.0×) (B37419AB, Beckman Coulter, Brea, CA, USA).

The original RNA data were submitted to the National Center of Biotechnology Information (NCBI) (www.ncbi.nlm.nih.gov/geo accessed on 5 April 2023) under the series entry PRJNA935509. Gene expression levels were standardized using the method of reads per kilobase million (RPKM) [103]. Genes with Fragments Per Kilobase of exon model per Million mapped fragments (FPKM) > 1 were considered expressed. Principal component analysis (PCA) was conducted using DESeq based on the sample expression levels, identifying genes that were differentially expressed with |log_2_ fold change| > 1 and *p* < 0.05 [104].

Statistical analysis of the sequencing data indicated that the percentage of Q30 bases in all libraries exceeded 90%, and the average guanine and cytosine (GC) content proportion of sequence bases before or after filtering in the clean data was over 43%, confirming the quality of the transcriptome data (Supplementary Table S3). The similarity between samples was high, as indicated by a correlation coefficient approaching 1, suggesting few differentially expressed genes between samples. Replicate samples demonstrated a correlation coefficient > 0.8, confirming good biological repeatability and reliable sequencing results (Supplementary Figure S8).

Clean reads were aligned to the peanut reference genome using HISAT2 (v2.0.5) software. Overall, the results mapped to the peanut genome samples were >89.61%, and the multiple mapping rates were <15.68%. The uniquely mapped segments of the reference genome were >76.63% (Supplementary Table S4). The transcriptome sequencing results met routine requirements, and the data were further analyzed.

#### 4.3.2. Weighted Correlation Network Analysis

To reveal the interrelationships between gene expression regulatory networks in peanuts under drought stress, weighted correlation network analysis (WGCNA) was used to construct a co-expression network. This analysis helped analyze the correlations between the modules and physiological and biochemical data, thereby enabling the identification of important genes associated with each characterization. The original transcription dataset of all samples was filtered to remove all genes with fragments per thousand bases (FPKM) of <1, even for a single repeat at any sampling point [105]. A scale-free co-expression network was constructed based on a soft threshold capacity of β = 8, which ensured that the adjacency matrix had a continuous value between 0 and 1, which was closer to the real biological network state. Second, a scale-free network was constructed using the clockwise module function. Gene co-expression modules were identified via module partitioning, and genes with similar expression were grouped. A dynamic tree-cutting algorithm was used to define the clustering tree-cutting components and assign them to different colors for visualization. The most important component of each module was calculated as a synthetic gene representing all gene expression in the module [106]. An interaction network of the seven gene modules was constructed using Cytoscape (https://cytoscape.org/ accessed on 3 June 2024).

#### 4.3.3. qRT-PCR Analysis

Eighteen differentially expressed genes (DEGs) were selected for qRT-PCR analysis to verify the RNA-Seq data (Supplementary Table S5). The peanut housekeeping actin gene (forward primer: TTGGAATGGGTCAGAAGGATGC; reverse primer: AGTGGTGCCTCAGTAAGAAGC) was used as an internal normalization reference and control in the same PCR cycle. First-strand cDNA was synthesized according to the manufacturer’s instructions using a SYBR Premix Ex Taq kit (TaKaRa, Beijing, China). The primers were designed by Probegene (Xuzhou, China) and synthesized by Shanghai Shenggong (Shanghai, China). Information about gene and primer sequences is presented in Supplementary Table S3. The qRT-PCR reaction system consisted of 0.1 μL of each primer (50 μM), 5 μL of the qRT-PCR mixture (2×), and 10 μL of DNA template (adjusted for DNA and cDNA concentrations), bringing the final volume to 20 μL with sterile distilled water. Each reaction included three biological replicates and three technical replicates per sample. The relative expressions were calculated using the 2^−ΔΔCT^ method [107]. The gene primer sequences are listed in Table S5. The reaction protocol was as follows: 95 °C for 10 min → 40 cycles → 95 °C for 15 s → 60 °C for 30 s, concluding with a melting curve analysis to confirm the specificity of the PCR [108].

### 4.4. Statistical Analysis

Statistical analysis was performed using GraphPad Prism 9.0, and data were expressed as mean ± standard deviation (SD). Univariate analysis of variance (ANOVA) using IBM SPSS Statistics 22 was performed to identify significant differences. The letters in the graph were significant at the 0.05 level of significance, with significant differences between letters in the same treatment time. Image-Pro Plus 6.0 (IPP 6.0) was used to measure tissue thickness in paraffin section images.

## 5. Conclusions

**Figure 16:**
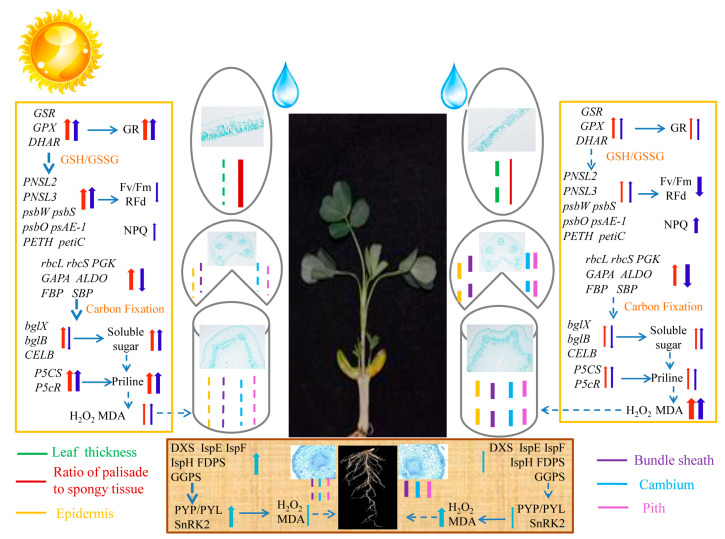
Regulation models of two peanut cultivars under acute drought stress during the seedling stage. Upward red arrows indicate positive effects on gene expression, physiology, and biochemistry in the stem. Upward blue arrows indicate positive effects on gene expression, physiology, and biochemistry in leaves; downward blue arrows indicate negative effects on gene expression, physiology, and biochemistry in leaves. Upward light blue arrows indicate positive effects on gene expression, physiology, and biochemistry in roots. The intense or weak font indicates strong and weak expression and physiological and biochemical changes under drought stress, as well as severe damage or light damage to the anatomical structure of the shoot and root. Bold red lines indicate an increased ratio of palisade to spongy tissue, whereas thin red lines indicate a decreased ratio. Dashed arrows indicate indirect effects, and arrows indicate activation.

In this study, two peanut cultivars differing in their tolerance to drought stress were evaluated under acute drought conditions. Both cultivars exhibited stress symptoms, including wilted leaves, bent stems, and reduced water contents in response to the drought; however, the decrease of water contents in the shoot of FH18 was more significant and continuously declined with prolonged drought stress. The accumulation of H_2_O_2_ and MDA was lower in HY22 than in FH18 (Figure 16). This trend was attributed to the SOD, POD, and GR activities being notably increased in HY22, which averted cell membrane lipid peroxidation and preserved the cell structure (Figure 16). Moreover, parameters related to photosynthesis were decreased in the leaves of both cultivars under acute drought stress, and gene expression related to photosynthesis, carbon fixation, and the CAM pathway was highly upregulated in the stem of HY22. Consequently, it was considered that increased amylase degradation by enzymes (bglX and bglB) provided a sufficient amount of soluble sugar to maintain osmosis (Figure 16). Furthermore, there was upregulated gene expression (*P5CS*) in the proline synthesis in HY22, resulting in higher proline levels than observed in FH18 (Figure 16). Photosynthesis in leaf as the initial photoautotroph system was severely damaged, but results show that the stem also played an important role in helping sustain photosynthesis (*psbW*, *psbB*, and *PETE*) and carbon fixation (*rbcL*, *PGK3*, *ALDO*, and *TKL*). In addition, other carbon fixation pathways such as CAM (*MODA* and *MDH1*) were activated (Figure 12). Those results provided a fresh insight into “cooperation response” in plants. Moreover, the root responses to drought stress, including terpenoid skeleton synthesis and the abscisic acid (ABA) transduction pathway, were notably increased in HY22, which promoted SOD and POD activity to clear away H_2_O_2_, thus maintaining a stable cell structure. Interestingly, under acute drought stress, shoot and root adopt different pathways and cooperate to maintain life activities.

## Figures and Tables

**Table 1:** Changes in peanut leaf tissue structures during the seedling stage under drought stress.

Drought Stress (h)	Leaf Thickness (μm)		Palisade Tissue Thickness (μm)		Spongy Tissue Thickness (μm)		Ratio of Palisade to Spongy Tissue	
	HY22	FH18	HY22	FH18	HY22	FH18	HY22	FH18
0	341.94 ± 8.72 a	358.82 ± 27.12 a	153.33 ± 2.57 c	150.56 ± 2.29 c	66.04 ± 2.10 a	62.43 ± 2.01 a	2.32 ± 0.09 c	2.41 ± 0.11 c
9	325.38 ± 10.42 ab	323.78 ± 8.80 ab	172.55 ± 2.35 a	166.58 ± 1.44 a	58.37 ± 1.21 c	58.23 ± 0.75 b	2.96 ± 0.05 a	2.86 ± 0.04 a
12	305.88 ± 15.76 b	293.70 ± 10.80 b	166.76 ± 1.42 b	159.87 ± 1.47 b	61.62 ± 0.74 b	61.29 ± 0.91 ab	2.71 ± 0.05 b	2.61 ± 0.02 b

Note: Different lowercase letters in the same column for each index indicate significant differences between samples (*p* < 0.05); mean ± standard error. “HY22” refers to the drought-tolerant cultivar Huayu 22, and “FH18” denotes the drought-sensitive cultivar Fuhua 18.

## Data Availability

All data analyzed during this study are provided in this published article and Supplementary Materials. The original sequencing data were submitted to the National Center of Biotechnology Information (NCBI) (www.ncbi.nlm.nih.gov/geo accessed on 5 April 2023) Sequence Read Archive (SRA) under accession number PRJNA935509.

## Supplementary Materials

The following supporting information can be downloaded at: https://www.mdpi.com/article/10.3390/ijms252211895/s1.
