# Supplementary material for: Transcriptome-Based Spatiotemporal Analysis of Drought Response Mechanisms in Two Distinct Peanut Cultivars
Source: Int J Mol Sci. 2024 Nov 5;25(22):11895. doi: 10.3390/ijms252211895 (PMC11593740; doi:10.3390/ijms252211895)
Supplement: Supplementary file 1 [file ijms-25-11895-s001.zip › 11-4 Supplementary figures and tables/Supplementary figures legends.docx]

Figure S1 Differentiated expression analyses of genes at seedling stage of peanuts under drought stress

(A) Differentially expressed gene change (B) (C) (D) Differentially expressed gene change in HY22 leaf (E) (F) (G) Differentially expressed gene change in FH18 leaf (H) (I) (J) Differentially expressed gene change in HY22 stem (K) (L) (M) Differentially expressed gene change in FH18 stem. “HY22” refers to the drought-tolerant cultivar Huayu 22, and “FH18” denotes the drought-sensitive cultivar Fuhua 18.

Figure S2 Correlation between qRT-PCR and RNA-seq based on their respective data from eight candidate transcripts

Each point represents the expression at 0 h, 9 h and 12 h. (A) HY22-*WBAI*; (B)HY22-*CLH2*; (C) HY22-*P5CS*; (D) HY22-*LACS8*; (E) HY22-*At3g13560*; (F) HY22 *asd*; (G) HY22-*lhcA*-P4; (H) HY22-*PNSL3*; “HY22” refers to the drought-tolerant cultivar Huayu 22.

Figure S3 Correlation between qRT-PCR and RNA-seq based on their respective data from eleven candidate transcripts

Each point represents the expression at 0 h, 9 h and 12 h. (I) FH18-*WBAI*; (J) FH18-*MYB15*; (K) FH18-*CHS*; (L) FH18-*CLH2*; (M) FH18- *GAPC2*; (N) FH18-*LACS8*; (O) FH18-*HSP83A*; (P) FH18-*asd*; (Q) FH18-*lhcA*-P4; (R) FH18-*P5CS*; (S) FH18-*PNSL3*; “FH18” denotes the drought-sensitive cultivar Fuhua 18.

Figure S4 Anatomical structures in different peanut cultivars under 9 and 15 days drought stress at the seedling stage (500 ×).

A. Leaf anatomical structures: a. upper epidermis; b. fence tissue; c. sponge tissue; d. water storage tissue; and e. epidermis. B. Stem anatomical structures: a. epidermis; b. vascular bundle sheath; c. vascular bundle cap; d. phloem; e. cambium; f. xylem; and g. medullary ray. C. Petiole anatomical structures: a. epidermis; b. vascular bundle sheath; c. vascular bundle cap; d. phloem; e. xylem; and f. medullary ray. “HY22” refers to the drought-tolerant cultivar Huayu 22, and “FH18” denotes the drought-sensitive cultivar Fuhua 18. “9D CK” denotes control of 9 day, and “15D CK” denotes control of 9 day.

Figure S5 Changes of stomatal opening of leaves of peanuts under drought stress at seedling stage（×40）

a Stomata; b. guard cells; c. accessory cell

Figure S6 The effect of 9 and 15 days’ drought stress on chlorophyll fluorescence parameters of leaves of different drought cultivars.

A. The ratio of maximum light quantum efficiency (Fv/Fm) of leaves of HY22 and FH18 after 9 and 15 days (D). B. The non-photochemical burst coefficient (NPQ) of leaves of HY22 and FH18 after 9 and 15 days (D). C. The variable fluorescence decline (Rfd) of leaves of HY22 and FH18 after 9 and 15 days (D). Values are means of three replicates, bars indicate ± standard error. Letters in graph are significant at 0.05 level of significance, with significant differ-ences between letters in the same treatment time. “HY22” refers to the drought-tolerant cultivar Huayu 22, and “FH18” denotes the drought-sensitive cultivar Fuhua 18.

Figure S7 Schematic diagram of sampling

Figure S8 Heat map of correlation between samples of peanut seedling under drought stress

“H-0-S”, “H-9-S”, and “H-12-S” refers to the stem of drought-tolerant cultivar Huayu 22 under 0, 9, and 12 h of drought stress, “H-0-L”, “H-9-L”, and “H-12-L” refers to the leaf of drought-tolerant cultivar Huayu 22 under 0, 9, and 12 h of drought stress. “F-0-S”, “F-9-S”, and “F-12-S” refers to the stem of drought-tolerant cultivar Fuhua 18 under 0, 9, and 12 h of drought stress, “F-0-L”, “F-9-L”, and “F-12-L” refers to the leaf of drought-tolerant cultivar Fuhua 18 under 0, 9, and 12 h of drought stress. “1, 2, and 3” indicated three replicates.
