# Supplementary material for: Transcriptome-Based Spatiotemporal Analysis of Drought Response Mechanisms in Two Distinct Peanut Cultivars
Source: Int J Mol Sci. 2024 Nov 5;25(22):11895. doi: 10.3390/ijms252211895 (PMC11593740; doi:10.3390/ijms252211895)
Supplement: Supplementary file 1 [file ijms-25-11895-s001.zip › 11-4 Supplementary figures and tables/Supplementary tables.docx]

Supplementary Table S1 RNA sample quality test results

| sample | Concentration  (ng/μL) | Volume (μL) | Total (μg) | Library type | Results |
| --- | --- | --- | --- | --- | --- |
| H-0-L-1 | 481 | 35 | 16.83 | transcriptome sequencing | A |
| H-0-L-2 | 353 | 35 | 12.35 | transcriptome sequencing | A |
| H-0-L-3 | 342 | 35 | 11.96 | transcriptome sequencing | A |
| H-9-L-1 | 378 | 35 | 13.23 | transcriptome sequencing | A |
| H-9-L-2 | 528 | 35 | 18.49 | transcriptome sequencing | A |
| H-9-L-3 | 315 | 35 | 11.03 | transcriptome sequencing | A |
| H-12-L-1 | 703 | 35 | 24.59 | transcriptome sequencing | A |
| H-12-L-2 | 683 | 35 | 23.90 | transcriptome sequencing | A |
| H-12-L-3 | 435 | 35 | 15.23 | transcriptome sequencing | A |
| F-0-L-1 | 478 | 35 | 16.74 | transcriptome sequencing | A |
| F-0-L-2 | 347 | 35 | 12.13 | transcriptome sequencing | A |
| F-0-L-3 | 788 | 35 | 27.58 | transcriptome sequencing | A |
| F-9-L-1 | 313 | 35 | 10.94 | transcriptome sequencing | A |
| F-9-L-2 | 305 | 35 | 10.66 | transcriptome sequencing | A |
| F-9-L-3 | 294 | 35 | 10.28 | transcriptome sequencing | A |
| F-12-L-1 | 297 | 35 | 10.40 | transcriptome sequencing | A |
| F-12-L-2 | 274 | 35 | 9.58 | transcriptome sequencing | A |
| F-12-L-3 | 426 | 35 | 14.93 | transcriptome sequencing | A |
| H-0-S-1 | 380 | 31 | 11.80 | transcriptome sequencing | A |
| H-0-S-2 | 185 | 40 | 7.39 | transcriptome sequencing | A |
| H-0-S-3 | 232 | 40 | 9.26 | transcriptome sequencing | A |
| H-9-S-1 | 139 | 35 | 4.85 | transcriptome sequencing | A |
| H-9-S-2 | 155 | 35 | 5.43 | transcriptome sequencing | A |
| H-9-S-3 | 183 | 35 | 6.41 | transcriptome sequencing | A |
| H-12-S-1 | 156 | 35 | 5.47 | transcriptome sequencing | A |
| H-12-S-2 | 200 | 35 | 6.99 | transcriptome sequencing | A |
| H-12-S-3 | 120 | 35 | 4.19 | transcriptome sequencing | A |
| F-0-S-1 | 272 | 35 | 8.44 | transcriptome sequencing | A |
| F-0-S-2 | 111 | 40 | 4.45 | transcriptome sequencing | A |
| F-0-S-3 | 157 | 40 | 6.26 | transcriptome sequencing | A |
| F-9-S-1 | 166 | 35 | 5.81 | transcriptome sequencing | A |
| F-9-S-2 | 168 | 35 | 5.88 | transcriptome sequencing | A |
| F-9-S-3 | 225 | 35 | 7.87 | transcriptome sequencing | A |
| F-12-S-1 | 156 | 35 | 5.45 | transcriptome sequencing | A |
| F-12-S-2 | 178 | 35 | 6.22 | transcriptome sequencing | A |
| F-12-S-3 | 235 | 35 | 8.21 | transcriptome sequencing | A |

Supplementary Table S3 Transcriptome sequencing raw data statistics analysis

| Sample | Raw Reads | Raw Bases | Clean Reads | Clean Bases | Q30 (%) | GC content (%) |
| --- | --- | --- | --- | --- | --- | --- |
| H-0-L-1 | 54728218 | 8209232700 | 54534038 | 8151438273 | 7572474698 (92.24%) | 3679868433 (44.83%) |
| H-0-L-2 | 43332726 | 6499908900 | 43190506 | 6457656206 | 6008247559 (92.44%) | 2911480056 (44.79%) |
| H-0-L-3 | 44659274 | 6698891100 | 44505642 | 6653615231 | 6145235061 (91.74%) | 2996628018 (44.73%) |
| H-9-L-1 | 50078114 | 7511717100 | 49919660 | 7464127252 | 6913370523 (92.03%) | 3342606115 (44.50%) |
| H-9-L-2 | 41318364 | 6197754600 | 41186450 | 6152270048 | 5691915442 (91.84%) | 2739043807 (44.19%) |
| H-9-L-3 | 51944388 | 7791658200 | 51773880 | 7742128683 | 7145439920 (91.71%) | 3452516472 (44.31%) |
| H-12-L-1 | 44748092 | 6712213800 | 44572500 | 6649379880 | 6209586390 (92.51%) | 2982186159 (44.43%) |
| H-12-L-2 | 52317254 | 7847588100 | 52118458 | 7769275136 | 7187009047 (91.58%) | 3472383883 (44.25%) |
| H-12-L-3 | 48388578 | 7258286700 | 48199430 | 7200686401 | 6632385509 (91.38%) | 3212848114 (44.26%) |
| F-0-L-1 | 46965852 | 7044877800 | 46816142 | 7003962038 | 6505297197 (92.34%) | 3150726536 (44.72%) |
| F-0-L-2 | 54528624 | 8179293600 | 54340870 | 8119548130 | 7498041331 (91.67%) | 3673972071 (44.92%) |
| F-0-L-3 | 57004816 | 8550722400 | 56811742 | 8494864082 | 7889592228 (92.27%) | 3846258672 (44.98%) |
| F-9-L-1 | 52189450 | 7828417500 | 52027920 | 7766898225 | 7233548727 (92.40%) | 3477419532 (44.42%) |
| F-9-L-2 | 50406312 | 7560946800 | 50232694 | 7503361472 | 6948441767 (91.90%) | 3352818737 (44.34%) |
| F-9-L-3 | 42167478 | 6325121700 | 42001020 | 6260700732 | 5818688661 (91.99%) | 2796090550 (44.21%) |
| F-12-L-1 | 50433564 | 7565034600 | 50252582 | 7501432544 | 6993970830 (92.45%) | 3345535255 (44.22%) |
| F-9-L-1 | 52189450 | 7828417500 | 52027920 | 7766898225 | 7233548727 (92.40%) | 3477419532 (44.42%) |
| F-9-L-2 | 50406312 | 7560946800 | 50232694 | 7503361472 | 6948441767 (91.90%) | 3352818737 (44.34%) |
| F-9-L-3 | 42167478 | 6325121700 | 42001020 | 6260700732 | 5818688661 (91.99%) | 2796090550 (44.21%) |
| F-12-L-1 | 50433564 | 7565034600 | 50252582 | 7501432544 | 6993970830 (92.45%) | 3345535255 (44.22%) |
| F-12-L-2 | 43357406 | 6503610900 | 43185968 | 6449776249 | 5958373454 (91.62%) | 2874038440 (44.19%) |
| F-12-L-3 | 48578282 | 7286742300 | 48408488 | 7236704093 | 6707926669 (92.06%) | 3208766423 (44.04%) |
| H-0-S-1 | 54154558 | 8123183700 | 53924046 | 8041369330 | 7429364815 (91.46%) | 3607360854 (44.41%) |
| H-0-S-2 | 45723596 | 6858539400 | 45552546 | 6797569389 | 6318879642 (92.13%) | 3024175396 (44.09%) |
| H-0-S-3 | 48329614 | 7249442100 | 48140192 | 7181263774 | 6638627934 (91.57%) | 3194163876 (44.06%) |
| H-9-S-1 | 49746656 | 7461998400 | 49574434 | 7395951660 | 6866971292 (92.03%) | 3359782663 (45.03%) |
| H-9-S-2 | 45907962 | 6886194300 | 45726684 | 6830796823 | 6355088465 (92.29%) | 3053591918 (44.34%) |
| H-9-S-3 | 41145562 | 6171834300 | 40974336 | 6102562571 | 5657263898 (91.66%) | 2756467227 (44.66%) |
| H-12-S-1 | 41172646 | 6175896900 | 41012728 | 6125562845 | 5664022127 (91.71%) | 2797275147 (45.29%) |
| H-12-S-2 | 50354264 | 7553139600 | 50151708 | 7490188866 | 6939078631 (91.87%) | 3415054936 (45.21%) |
| H-12-S-3 | 43056552 | 6458482800 | 42887242 | 6407660994 | 5928929294 (91.80%) | 2903099643 (44.95%) |
| F-0-S-1 | 48102802 | 7215420300 | 47917052 | 7130540267 | 6639843326 (92.02%) | 3172416734 (43.97%) |
| F-0-S-2 | 52097702 | 7814655300 | 51912482 | 7737557358 | 7221656017 (92.41%) | 3432826937 (43.93%) |
| F-0-S-3 | 43898188 | 6584728200 | 43749918 | 6521217523 | 6089136274 (92.47%) | 2895350225 (43.97%) |
| F-9-S-1 | 41856176 | 6278426400 | 41689378 | 6226549293 | 5753721409 (91.64%) | 2796935659 (44.55%) |
| F-9-S-2 | 55232912 | 8284936800 | 55028744 | 8215535359 | 7610514168 (91.86%) | 3694657070 (44.59%) |
| F-9-S-3 | 50586788 | 7588018200 | 50403418 | 7521810846 | 6985500101 (92.06%) | 3367552992 (44.38%) |
| F-12-S-1 | 53933712 | 8090056800 | 53718248 | 8033469776 | 7393935702 (91.40%) | 3616724207 (44.71%) |
| F-12-S-2 | 44692136 | 6703820400 | 44509758 | 6650301437 | 6091024049 (90.86%) | 2992176582 (44.63%) |
| F-12-S-3 | 48310590 | 7246588500 | 48138526 | 7198654332 | 6671478760 (92.06%) | 3240104504 (44.71%) |

Supplementary Table S4 Statistics of comparison between the sample and the peanut reference genome

| Sample | Clean Reads | Total mapped | Multiple mapped | Unique mapped |
| --- | --- | --- | --- | --- |
| H-0-Y-1 | 54534038 | 52955808(97.39%) | 7495920(13.79%) | 45459888(83.61%) |
| H-0-Y-2 | 43190506 | 42079923(97.83%) | 6192039(14.39%) | 35887884(83.43%) |
| H-0-Y-3 | 44505642 | 43179068(97.40%) | 6332160(14.28%) | 36846908(83.12%) |
| H-9-Y-1 | 49919660 | 48268313(97.24%) | 7445917(15.00%) | 40822396(82.24%) |
| H-9-Y-2 | 41186450 | 38213472(92.97%) | 5505217(13.39%) | 32708255(79.58%) |
| H-9-Y-3 | 51773880 | 50288772(97.41%) | 7246216(14.04%) | 43042556(83.37%) |
| H-12-Y-1 | 44572500 | 43307491(97.53%) | 6471258(14.57%) | 36836233(82.96%) |
| H-12-Y-2 | 52118458 | 50598674(97.29%) | 7797939(14.99%) | 42800735(82.30%) |
| H-12-Y-3 | 48199430 | 46659586(97.28%) | 6793223(14.16%) | 39866363(83.12%) |
| F-0-Y-1 | 46816142 | 45434183(97.62%) | 6556843(14.09%) | 38877340(83.53%) |
| F-0-Y-2 | 54340870 | 52683286(97.37%) | 7904975(14.61%) | 44778311(82.76%) |
| F-0-Y-3 | 56811742 | 55329775(97.69%) | 7780947(13.74%) | 47548828(83.95%) |
| F-9-Y-1 | 52027920 | 50798272(97.89%) | 7497352(14.45%) | 43300920(83.44%) |
| F-9-Y-2 | 50232694 | 48853209(97.48%) | 7374463(14.72%) | 41478746(82.77%) |
| F-9-Y-3 | 42001020 | 40877859(97.54%) | 6163834(14.71%) | 34714025(82.83%) |
| F-12-Y-1 | 50252582 | 48793430(97.66%) | 7402249(14.82%) | 41391181(82.85%) |
| F-12-Y-2 | 43185968 | 41897156(97.27%) | 6131342(14.24%) | 35765814(83.04%) |
| F-12-Y-3 | 48408488 | 43257022(89.61%) | 6265906(12.98%) | 36991116(76.63%) |
| H-0-J-1 | 53924046 | 52318673(97.15%) | 7742767(14.38%) | 44575906(82.77%) |
| H-0-J-2 | 45552546 | 44364196(97.50%) | 6440823(14.16%) | 37923373(83.35%) |
| H-0-J-3 | 48140192 | 46657538(97.06%) | 7019056(14.60%) | 39638482(82.46%) |
| H-9-J-1 | 49574434 | 48158599(97.62%) | 7339228(14.88%) | 40819371(82.74%) |
| H-9-J-2 | 45726684 | 44137765(96.99%) | 6412940(14.09%) | 37724825(82.90%) |
| H-9-J-3 | 40974336 | 39660434(97.36%) | 6098473(14.97%) | 33561961(82.39%) |
| H-12-J-1 | 41012728 | 39059860(97.03%) | 6269680(15.57%) | 32790180(81.45%) |
| H-12-J-2 | 50151708 | 48289587(97.33%) | 7779373(15.68%) | 40510214(81.65%) |
| H-12-J-3 | 42887242 | 41382374(97.41%) | 6308431(14.85%) | 35073943(82.56%) |
| F-0-J-1 | 47917052 | 46632445(97.44%) | 6863013(14.34%) | 39769432(83.10%) |
| F-0-J-2 | 51912482 | 50380237(97.17%) | 7493029(14.45%) | 42887208(82.72%) |
| F-0-J-3 | 43749918 | 42582978(97.45%) | 6212679(14.22%) | 36370299(83.23%) |
| F-9-J-1 | 41689378 | 40361548(97.31%) | 5807254(14.00%) | 34554294(83.31%) |
| F-9-J-2 | 55028744 | 53412613(97.44%) | 7949964(14.50%) | 45462649(82.94%) |
| F-9-J-3 | 50403418 | 48989010(97.45%) | 7168768(14.26%) | 41820242(83.19%) |
| F-12-J-1 | 53718248 | 51189404(95.79%) | 7407627(13.86%) | 43781777(81.92%) |
| F-12-J-2 | 44509758 | 42978728(96.85%) | 6099684(13.75%) | 36879044(83.10%) |
| F-12-J-3 | 48138526 | 46764886(97.55%) | 6919940(14.44%) | 39844946(83.12%) |

Note: Number of Clean Reads and high quality reads; Total mapped, the number of all reads that could be mapped to the genome and the proportion of effective reads; Multiple mapped: The number of reads in the reference genome and the proportion of effective reads in multiple comparisons; Unique mapped, the number of reads and the proportion of valid reads in the reference genome.

Supplementary Table S5 Primer sequences for candidate reference genes

| id | Symbol | Description | (F) Sense primer ( 5’- 3’) | | (R)Antisense primer (5’- 3’) |
| --- | --- | --- | --- | --- | --- |
| *arahy.Tifrunner.gnm2.ann1.27FFWB* | WBAI | QHO58714.1 Seed lectin [*Arachis hypogaea*] | | CAGCCTCGGCAGAGTTAC | GATTTGTTTGGAGTGTTGATG |
| *arahy.Tifrunner.gnm2. ann1. RQXR90* | CLH2 | XP_025635176.1 chlorophyllase-2, chloroplastic-like [*Arachis hypogaea*] | | GGTGGCTGGACCCGATAC | GCCGAAAACCTGAGATTAGTG |
| *arahy.Tifrunner.gnm2. ann1. WS4P7I* | P5CS | XP_016183778.1 delta-1-pyrroline-5-carboxylate synthase isoform X2 [*Arachis ipaensis*] | | GATACTAAGAGGAAATGGTCAGG | GGCAGCATCTCAGCAACT |
| *arahy.Tifrunner.gnm2. ann1.EW431R* | LACS8 | XP_016175183.1 long chain acyl-CoA synthetase 8 [*Arachis ipaensis*] | | GGGCATCAGTATCTTACCACG | CGGAATCACAACCGAACC |
| *arahy.Tifrunner.gnm2. ann1.5E5ZG3* | At3g13560 | RYR11675.1 hypothetical protein Ahy_B04g069194 [*Arachis hypogaea*] | | ACAAGAGGAATGGACCGA | CCACTAAAACTCAAAGGATAAAC |
| *arahy.Tifrunner.gnm2. ann1.GF1W8W* | asd | XP_016190828.1 uncharacterized protein LOC107631792 [*Arachis ipaensis*] | | CATCAGCAAGGAGTTCGG | GCATGGCTTCAGGGTTCA |
| *arahy.Tifrunner.gnm2. ann1.RLTX4G* | lhcA-P4 | XP_015937861.1 chlorophyll a-b binding protein P4, chloroplastic [*Arachis duranensis*] | | TTCCGAAATGGTACGACG | TCCCAGGGTTCTTGATGT |
| *arahy.Tifrunner.gnm2. ann1.IUT8LB* | PNSL3 | XP_025614717.1 photosynthetic NDH subunit of lumenal location 3, chloroplastic [*Arachis hypogaea*] | | CTTGTTGCCTCCACAGTTC | CATCATACCACCAGCCATT |
| *arahy.Tifrunner.gnm2.ann1.27FFWB* | WBAI | QHO58714.1 Seed lectin [*Arachis hypogaea*] | | CAGCCTCGGCAGAGTTAC | GATTTGTTTGGAGTGTTGATG |
| *arahy.Tifrunner.gnm2.ann1.H3HGUT* | MYB15 | XP_016176474.1 myb-related protein Myb4 [*Arachis ipaensis*] | | TGGTGGGGTCCAGTGTCA | CAGAATACCTAGATTTTGTCGC |
| *arahy.Tifrunner.gnm2. ann1. NS7LJX* | CHS | XP_025694419.1 stilbene synthase 1-like [*Arachis hypogaea*] | | AACTCATCGTACTCTTAGGACTC | CTCACTAGGACCACGGAAA |
| *arahy.Tifrunner.gnm2. ann1. VRBE94* | GAPC2 | XP_016169919.1 glyceraldehyde-3-phosphate dehydrogenase GAPC2, cytosolic [*Arachis ipaensis*] | | AATGGCTTTCCGTGTTCC | CGTCATAGGTGGCAGGCT |
| *arahy.Tifrunner.gnm2. ann1. RQXR90* | CLH2 | XP_025635176.1 chlorophyllase-2, chloroplastic-like [*Arachis hypogaea*] | | GGTGGCTGGACCCGATAC | GCCGAAAACCTGAGATTAGTG |
| *arahy.Tifrunner.gnm2. ann1. VRBE94* | GAPC2 | XP_016169919.1 glyceraldehyde-3-phosphate dehydrogenase GAPC2, cytosolic [*Arachis ipaensis*] | | AATGGCTTTCCGTGTTCC | CGTCATAGGTGGCAGGCT |
| *arahy.Tifrunner.gnm2.ann1.H3HGUT* | MYB15 | XP_016176474.1 myb-related protein Myb4 [*Arachis ipaensis*] | | TGGTGGGGTCCAGTGTCA | CAGAATACCTAGATTTTGTCGC |
| *arahy.Tifrunner.gnm2. ann1. NS7LJX* | CHS | XP_025694419.1 stilbene synthase 1-like [*Arachis hypogaea*] | | AACTCATCGTACTCTTAGGACTC | CTCACTAGGACCACGGAAA |
| *arahy.Tifrunner.gnm2. ann1.L5T5KA* | HSP83A | XP_016189472.1 heat shock protein 83 [*Arachis ipaensis*] | | TTCGGTGTTGGCTTCTACT | GGTGATCTTGGTGCCTCTA |
| *arahy.Tifrunner.gnm2. ann1.EW431R* | LACS8 | XP_016175183.1 long chain acyl-CoA synthetase 8 [*Arachis ipaensis*] | | GGGCATCAGTATCTTACCACG | CGGAATCACAACCGAACC |
| *arahy.Tifrunner.gnm2. ann1.L5T5KA* | HSP83A | XP_016189472.1 heat shock protein 83 [*Arachis ipaensis*] | | TTCGGTGTTGGCTTCTACT | GGTGATCTTGGTGCCTCTA |
| *arahy.Tifrunner.gnm2. ann1.GF1W8W* | asd | XP_016190828.1 uncharacterized protein LOC107631792 [*Arachis ipaensis*] | | CATCAGCAAGGAGTTCGG | GCATGGCTTCAGGGTTCA |
| *arahy.Tifrunner.gnm2. ann1.RLTX4G* | lhcA-P4 | XP_015937861.1 chlorophyll a-b binding protein P4, chloroplastic [*Arachis duranensis*] | | TTCCGAAATGGTACGACG | TCCCAGGGTTCTTGATGT |
| *arahy.Tifrunner.gnm2. ann1.1W477Y* | P5CS | XP_025695370.1 delta-1-pyrroline-5-carboxylate synthase isoform X2 [*Arachis hypogaea*] | | AAGACCCTGAAGTTGCTGA | ATCTGTTTTATTTGTCGTTGTG |
| *arahy.Tifrunner.gnm2. ann1.IUT8LB* | PNSL3 | XP_025614717.1 photosynthetic NDH subunit of lumenal location 3, chloroplastic [*Arachis hypogaea*] | | CTTGTTGCCTCCACAGTTC | CATCATACCACCAGCCATT |
