# Supplementary figures and images for: Transcriptome-Based Spatiotemporal Analysis of Drought Response Mechanisms in Two Distinct Peanut Cultivars
Source: Int J Mol Sci. 2024 Nov 5;25(22):11895. doi: 10.3390/ijms252211895 (PMC11593740; doi:10.3390/ijms252211895)

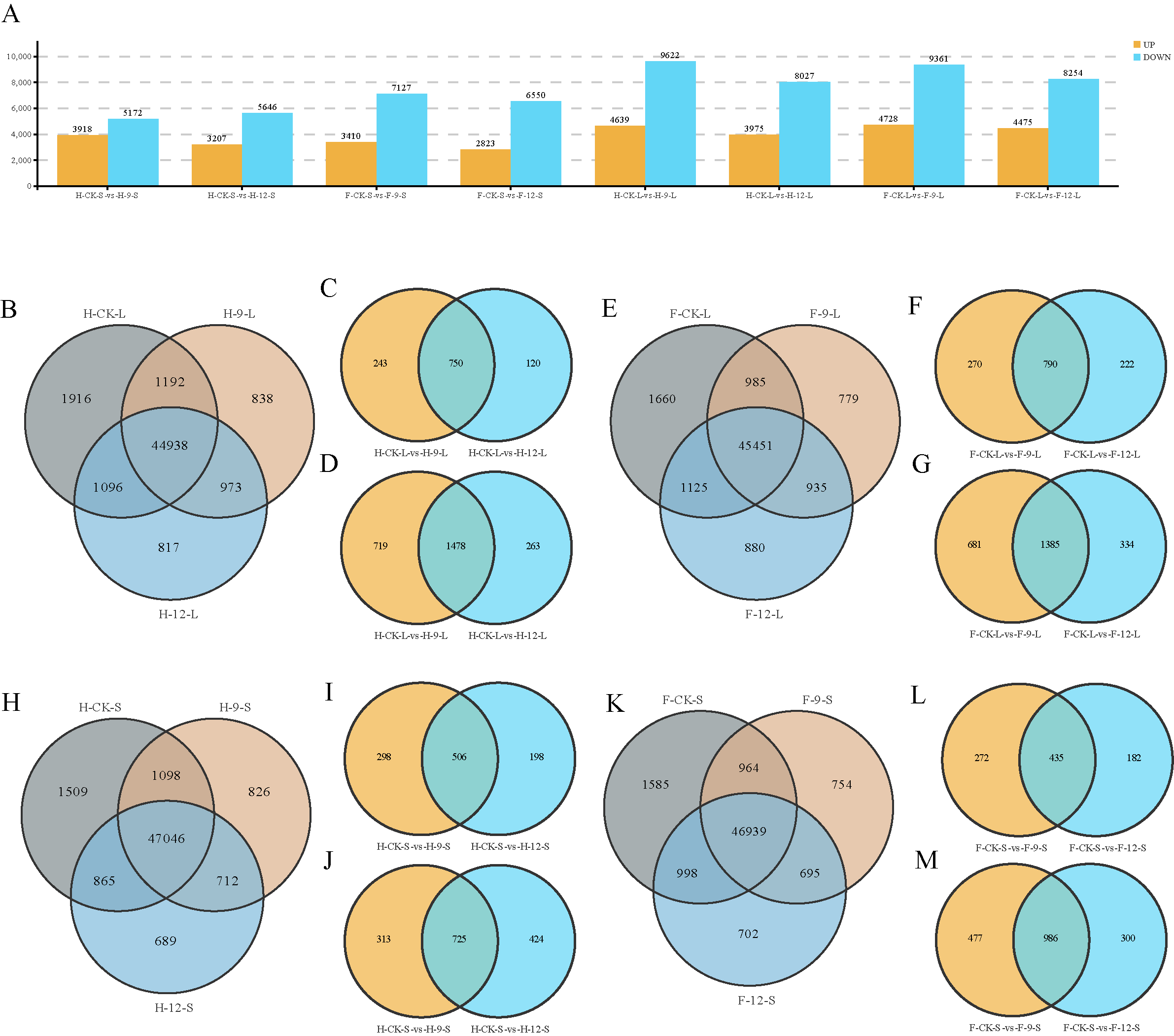

Supplement: Supplementary file 1 [file ijms-25-11895-s001.zip › 11-4 Supplementary figures and tables/Figure S1 Differentiated expression analyses of genes at seedling stage of peanuts under drought stress.tif]

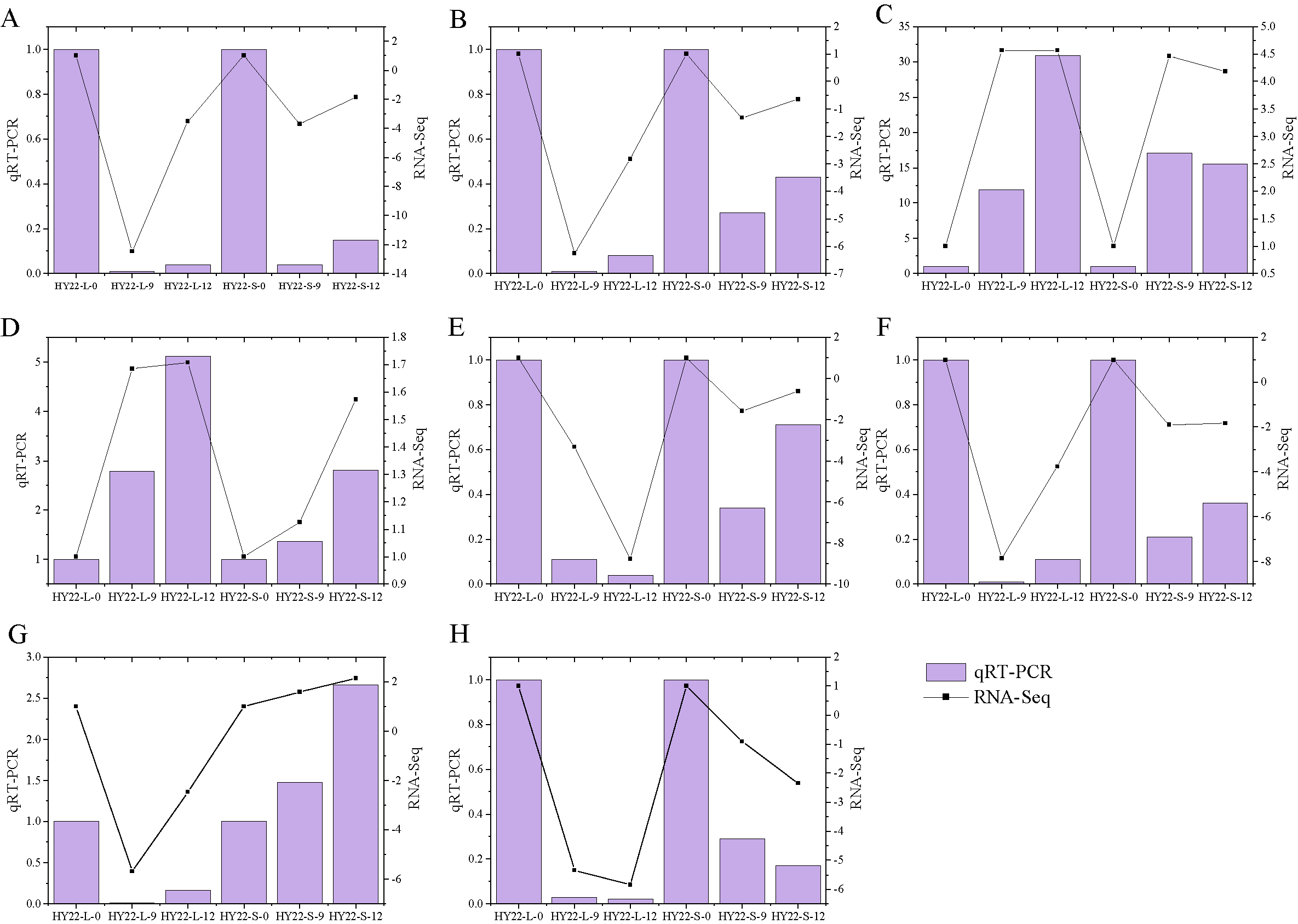

Supplement: Supplementary file 1 [file ijms-25-11895-s001.zip › 11-4 Supplementary figures and tables/Figure S2 Correlation between qRT-PCR and RNA-seq based on their respective data.tif]

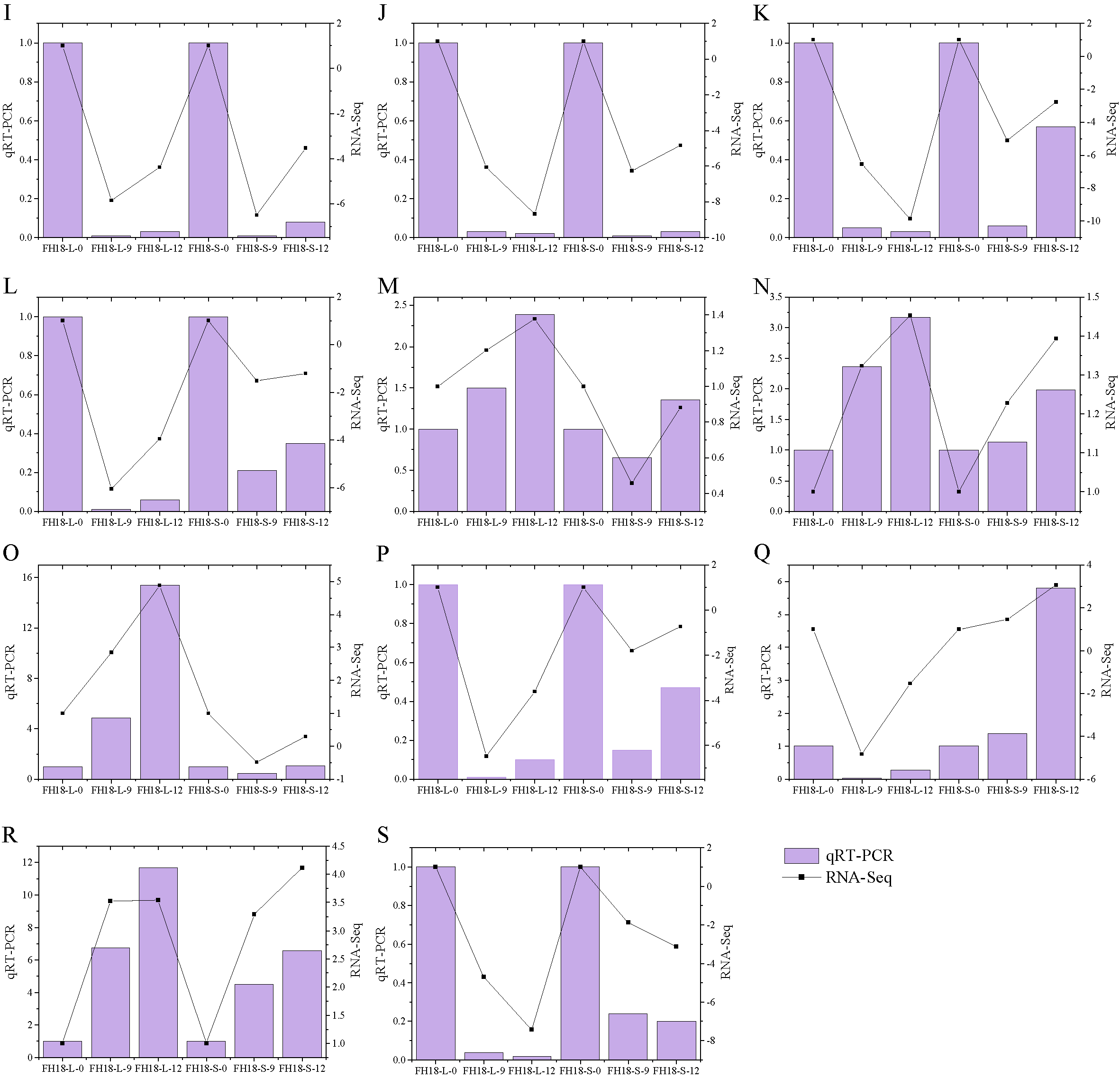

Supplement: Supplementary file 1 [file ijms-25-11895-s001.zip › 11-4 Supplementary figures and tables/Figure S3 Correlation between qRT-PCR and RNA-seq based on their respective data.tif]

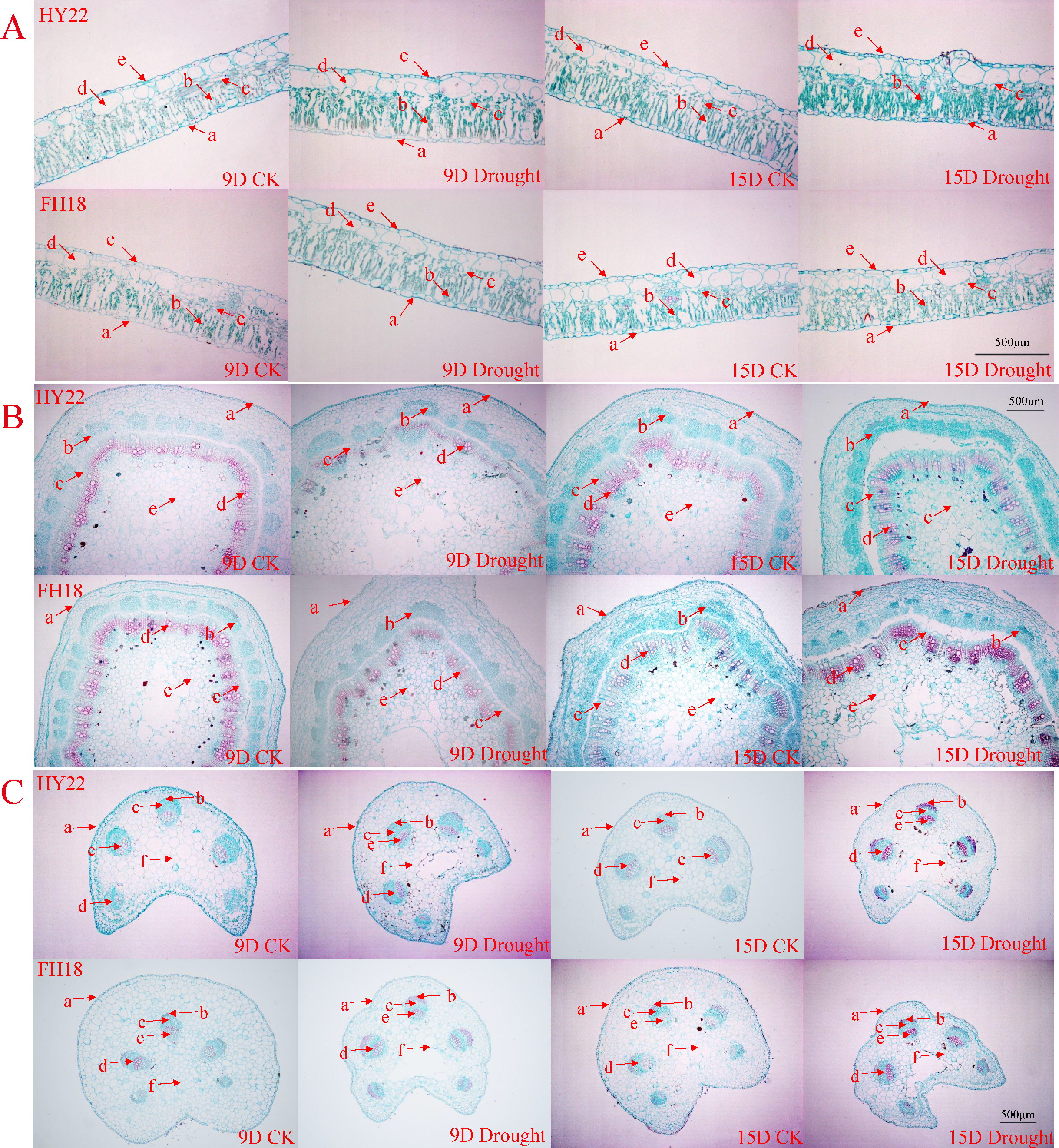

Supplement: Supplementary file 1 [file ijms-25-11895-s001.zip › 11-4 Supplementary figures and tables/Figure S4 Anatomical structures in different peanut cultivars under 9 and 15 days drought stress at the seedling stage (500 ×)..tif]

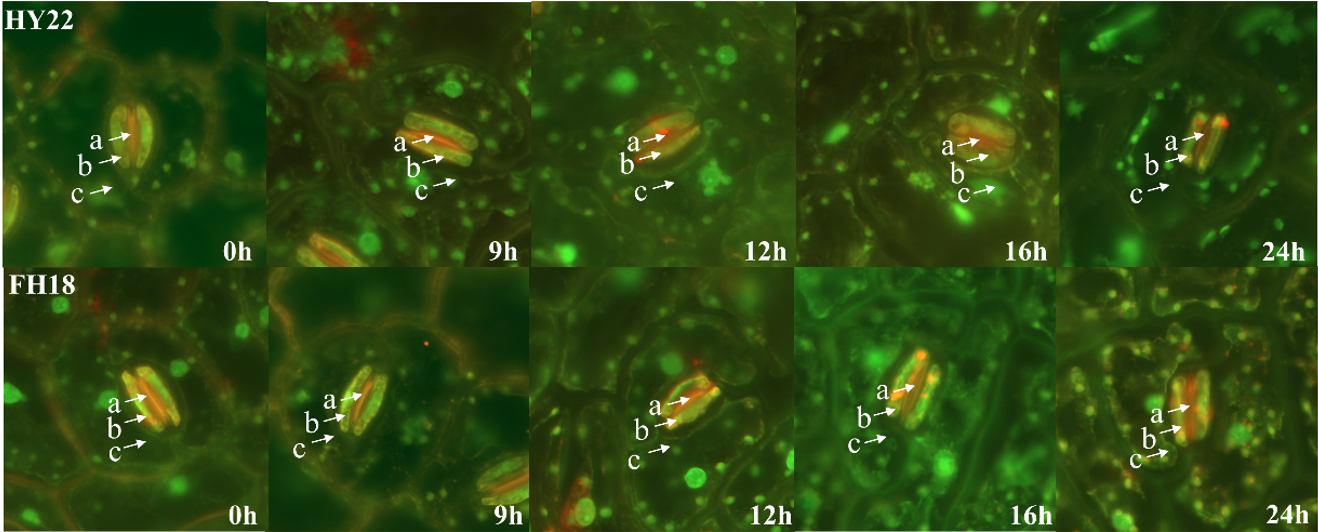

Supplement: Supplementary file 1 [file ijms-25-11895-s001.zip › 11-4 Supplementary figures and tables/Figure S5 Changes of stomatal opening of leaves of peanuts under drought stress at seedling stage(×40)a Stomata; b. guard cells; c. accessory cell.png]

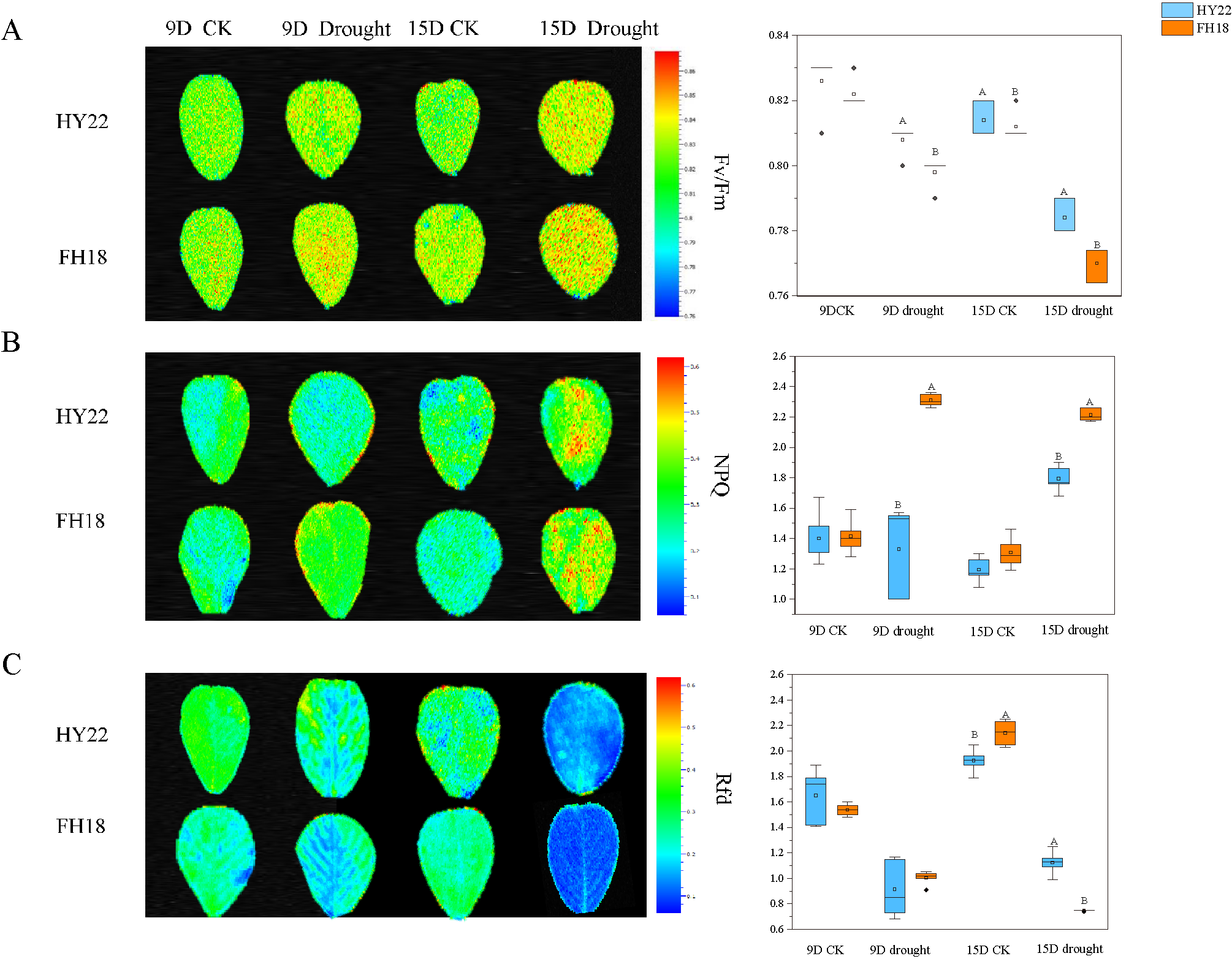

Supplement: Supplementary file 1 [file ijms-25-11895-s001.zip › 11-4 Supplementary figures and tables/Figure S6 The effect of 9 and 15 days drought stress on chlorophyll fluorescence parameters of leaves of different drought cultivars..tif]

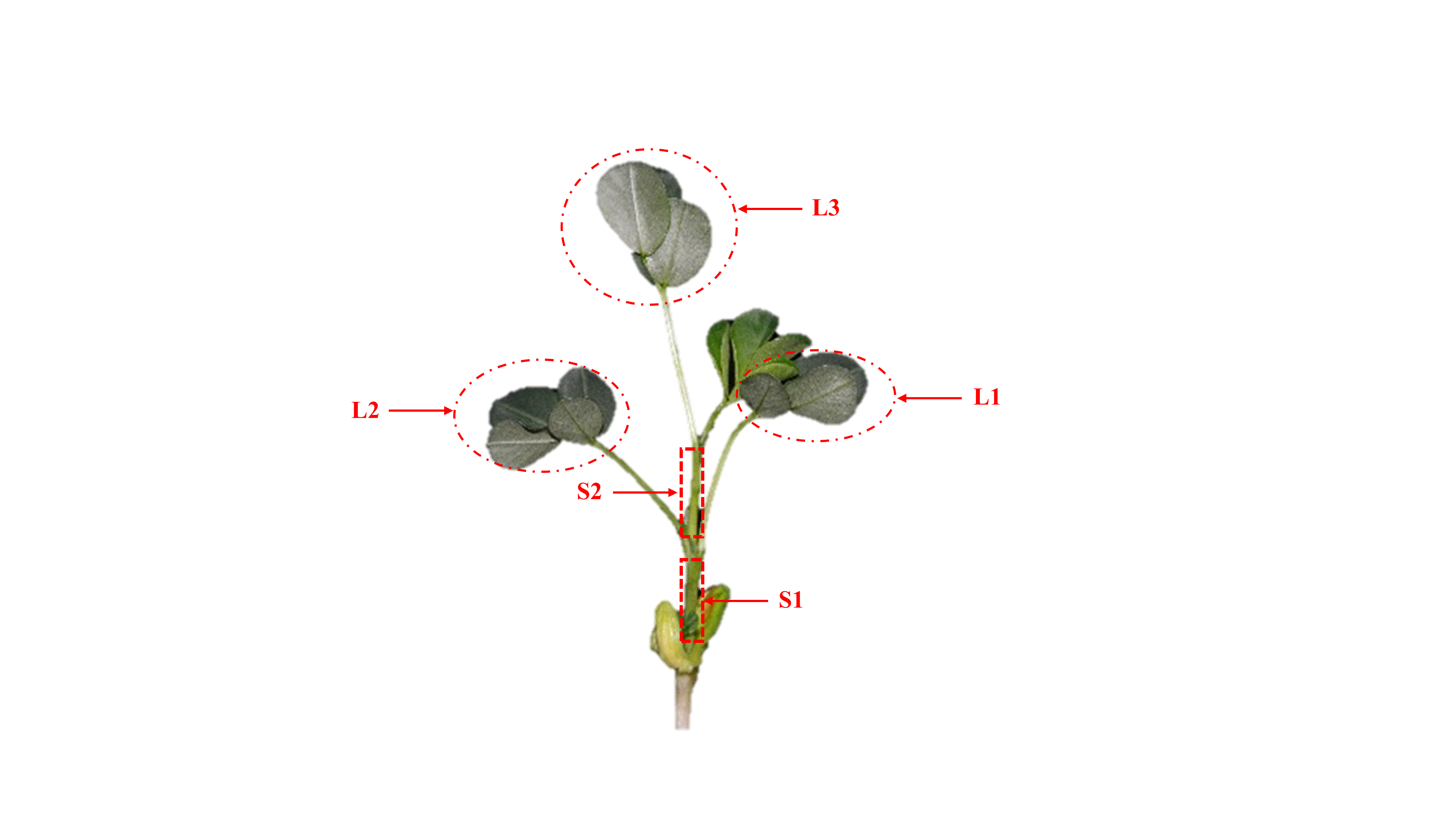

Supplement: Supplementary file 1 [file ijms-25-11895-s001.zip › 11-4 Supplementary figures and tables/Figure S7 Schematic diagram of sampling.png]

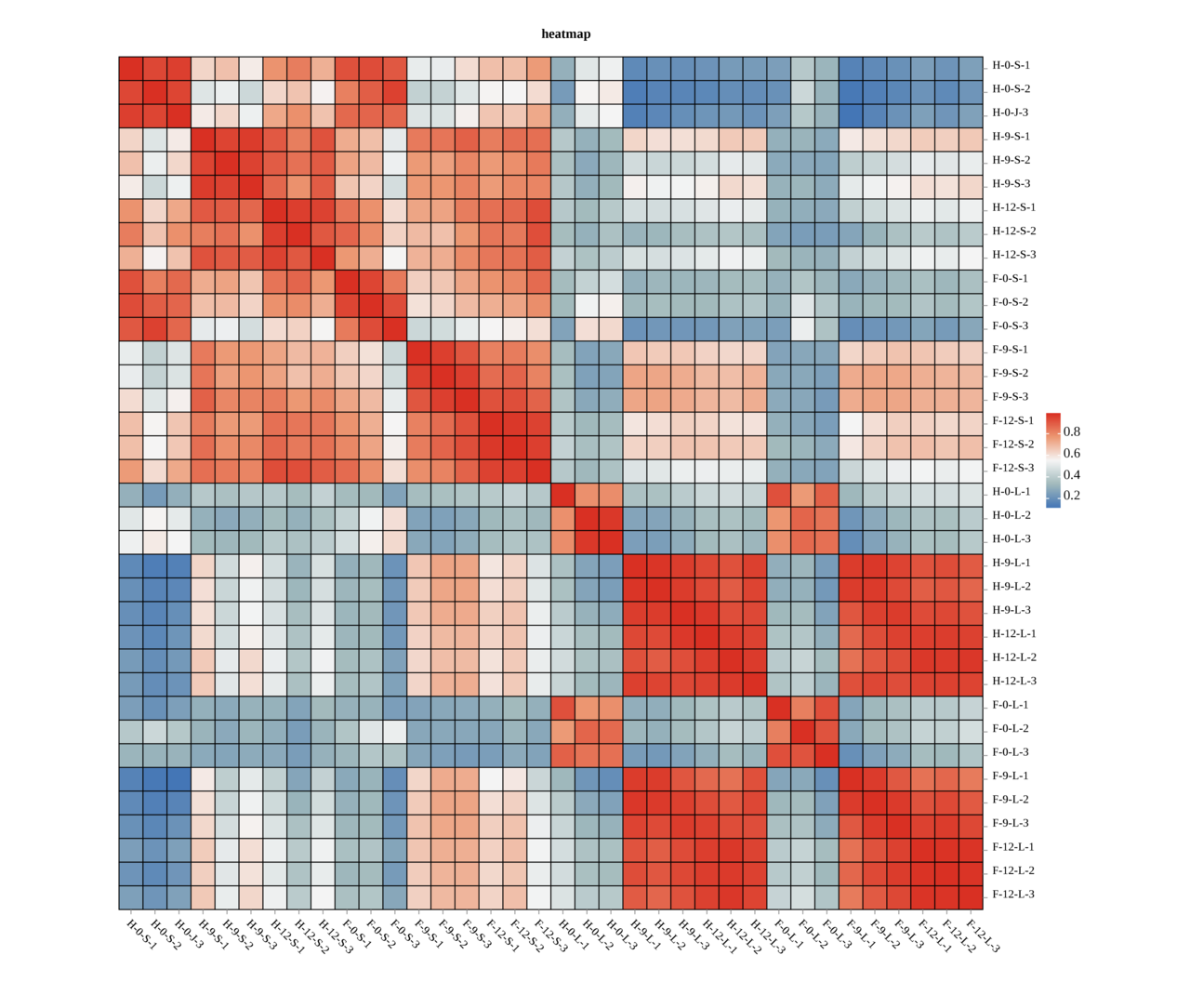

Supplement: Supplementary file 1 [file ijms-25-11895-s001.zip › 11-4 Supplementary figures and tables/Figure S8 Heat map analysis of correlation between samples of peanut seedlings under drought stress.tiff]
